# Spectrum projection with a bandgap-gradient perovskite cell for colour perception

**DOI:** 10.1038/s41377-020-00400-w

**Published:** 2020-09-15

**Authors:** Mei-Na Zhang, Xiaohan Wu, Antoine Riaud, Xiao-Lin Wang, Fengxian Xie, Wen-Jun Liu, Yongfeng Mei, David Wei Zhang, Shi-Jin Ding

**Affiliations:** 1 State Key Laboratory of ASIC and System, School of Microelectronics, Fudan University, Shanghai, 200433 China; 2 Engineering Research Centre of Advanced Lighting Technology, Ministry of Education, Fudan University, Shanghai, 200433 China; 3 Department of Materials Science, Fudan University, Shanghai, 200433 China

**Keywords:** Optoelectronic devices and components, Imaging and sensing

## Abstract

Optoelectronic devices for light or spectral signal detection are desired for use in a wide range of applications, including sensing, imaging, optical communications, and in situ characterization. However, existing photodetectors indicate only light intensities, whereas multiphotosensor spectrometers require at least a chip-level assembly and can generate redundant signals for applications that do not need detailed spectral information. Inspired by human visual and psychological light perceptions, the compression of spectral information into representative intensities and colours may simplify spectrum processing at the device level. Here, we propose a concept of spectrum projection using a bandgap-gradient semiconductor cell for intensity and colour perception. Bandgap-gradient perovskites, prepared by a halide-exchanging method via dipping in a solution, are developed as the photoactive layer of the cell. The fabricated cell produces two output signals: one shows linear responses to both photon energy and flux, while the other depends on only photon flux. Thus, by combining the two signals, the single device can project the monochromatic and broadband spectra into the total photon fluxes and average photon energies (i.e., intensities and hues), which are in good agreement with those obtained from a commercial photodetector and spectrometer. Under changing illumination in real time, the prepared device can instantaneously provide intensity and hue results. In addition, the flexibility and chemical/bio-sensing of the device via colour comparison are demonstrated. Therefore, this work shows a human visual-like method of spectrum projection and colour perception based on a single device, providing a paradigm for high-efficiency spectrum-processing applications.

## Optoelectronics: photodetectors capable of perceiving light intensity and colour

Chinese scientists have made a low cost, flexible photodetector that can detect light intensity and perceive colour, laying the foundations for new devices for use in a wide range of applications, including sensing, imaging, and optical communications. Current optoelectronic detectors are only able to perceive light intensities. Although multiphotosensor spectrometers are capable of perceiving intensity and colour, they require chip-level assembly and can generate redundant signals for applications that do not require detailed information. Now, S.-J.D. and colleagues from Fudan University in China have created a photodetector made from a single semiconductor bandgap-gradient perovskite cell. The device consists of a photoactive layer made from a semiconductor with a small bandgap and a photosensing layer made from a semiconductor with gradient bandgaps and is capable of detecting light intensity and perceiving colour.

## Introduction

Optoelectronic devices that provide light responses and spectral measurements are promising components for use in a wide range of applications, including multifunctional sensing, colour imaging, high-bandwidth optical communications, and in situ characterization techniques^1–14^. However, the current selection of optoelectronic detectors is used to measure only light intensities (power densities, *p*)^1–9^. Most photodetectors can be classified into thermal and semiconductive types according to their working principles. The former, combining thermopiles with light absorbers, converts light to heat and then to electrical signals indirectly indicating light power densities^10–12^. The latter, directly converting photons to excitons, provides electrical signals depending on the photon flux *φ* and energy *ε*^2,7,8^. For a constant *φ*, the response of a semiconductor to *ε* is dependent on its light absorption *A*(*ε*) and photon energy-dependent quantum efficiency *η*(*ε*) for the absorbed photons. Both *A*(*ε*) and *η*(*ε*) exhibit fluctuating curves for semiconductors with indirect bandgaps (e.g., silicon) and step-like curves for those with direct bandgaps. This means that semiconductive photodetectors either give fluctuating responses to *ε* or provide only limited information about *ε*. Ordinary semiconductive photodetectors can only output light power densities by combining photon flux and energy (*p* = *εφ*) after calibration, and most often, the user must input the current wavelength (e.g., commercial silicon photodiodes). Moreover, a narrowband photodetector is only sensitive to specific lights over a rather small spectral range;^3,13^ these narrowband photodetectors generally exhibit indiscriminate sensitivity to lights over their detectable spectral range and are blind to the spectrum beyond their detectable wavelengths. Thus, none of the existing photodetectors are able to provide spectral information at the device level. It was recently reported that integrating dozens or hundreds of photodetectors with semiconductors presenting different bandgaps can reconstruct the spectral curves of incident light^14,15^. Nevertheless, such an approach already requires a chip-level device assembly and signal-processing system, and can generate redundant signals for applications that do not need detailed spectral information.

Our brain perceives light as a combination of colours and intensities, which allows it to process a large number of light signals with high efficiency^16–19^. Inspired by this, the projection of spectra into representative indices may enable true and efficient spectrum processing at the device level. Studies on colour perception suggest that while the human eye perceives three distinct wavelengths (red, green, and blue), the human brain projects the colours in terms of hue and intensity, where the colour is best represented by the hue^16,18,20^. Therefore, a device for spectrum processing and colour perception should be capable of the following: (1) discriminatively collect a light signal in different spectral regions—the more signals from different spectral regions, the higher the hue resolution is and (2) output compressed electrical signals directly indicating the hue and intensity of the incident light.

In this work, we propose a concept of spectrum projection and colour perception through the use of a single device by introducing bandgap-gradient semiconductors as photoactive materials. Since photon absorption can only occur at energies above the bandgap of semiconductors, the devices can sense the spectral content of light signals with high resolution. Halide perovskite films with a continuous gradient of direct bandgaps are developed to demonstrate a device that outputs a signal exhibiting linear responses to both *ε* and *φ*. Combining the other output signal that is linearly dependent only on *φ*, the intensity and hue (i.e., total photon flux and average photon energy) of spectra can be obtained from a single device. The mechanical flexibility of the devices is demonstrated by fabricating them on polymer substrates. The online colour perception is shown by using the device to sense real-time changes in lighting. Finally, sensing applications are demonstrated by using the device to perceive the colour changes of stimuli-sensitive materials that are responsive to chemical/bio-analytes.

## Results

### Design of colour-perception device

**Fig. 1:**
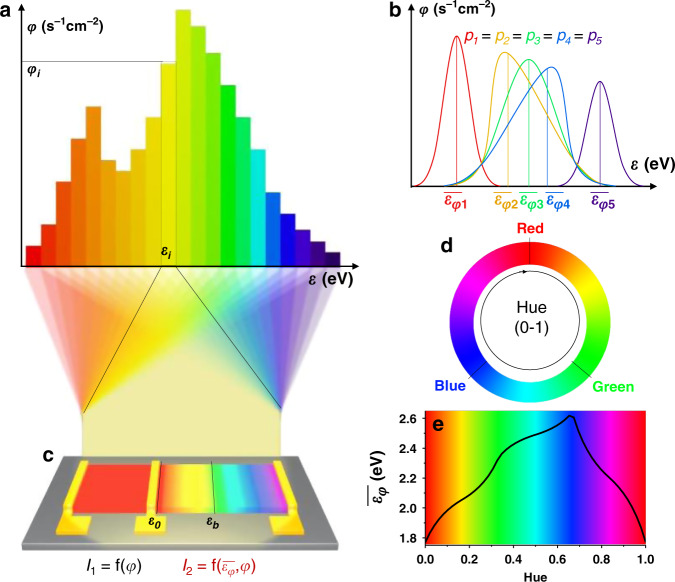
Conceptual design of colour perception using a single device. **a** Spectral diagram. **b** Scheme of the average photon energy. **c** Colour perception using an optoelectronic device with bandgap-gradient semiconductors, in which one output signal is determined only by the total photon flux (*I*_1_ = f (*φ*)), and the other is dependent on both the total photon flux and average photon energy ($I_2 = {\mathrm{f}}\left( {\overline {\varepsilon _\varphi } ,\varphi } \right)$). **d** Scheme of human visual colour perception in terms of hue (value 0–1). **e** Correlation between hue and average photon energy.

Figure 1a shows the diagram of a spectrum containing photons with different energies. The photon flux for a given photon energy *ε*_*i*_ is defined as *φ*_*i*_, and the total power density is given by $p = \mathop {\sum}\nolimits_i {\varepsilon _i\varphi _i}$. Statistically, representative indices for a spectrum should include the total photon flux $\varphi = \mathop {\sum}\nolimits_i {\varphi _i}$ and the average photon energy weighted by the flux $\bar \varepsilon _\varphi = \mathop {\sum}\nolimits_i {\varepsilon _i\varphi _i} /\mathop {\sum}\nolimits_i {\varphi _i = p/\varphi }$. Such indices are critical to spectrum identifications; that is, $\bar \varepsilon _\varphi$ can be clearly different for the spectra even with the same *p*, as illustrated in Fig. 1b. An ideal colour-perception device should be able to discriminatively absorb the photon flux corresponding to each photon energy in the spectrum and project them into output signals that directly indicate $\bar \varepsilon _\varphi$ and *φ*. Accordingly, we propose the device design shown in Fig. 1c. The device consists of two parts and outputs two signals, in which one part uses a semiconductor with a rather small bandgap (SBG) as a photoactive layer and exhibits an output current (*I*_*1*_) determined only by *φ* in a certain *ε* region. The other part is composed of semiconductors with gradient bandgaps (GBGs) as a photosensing layer, and a high photon energy *ε* can lead to a large photoexcited domain (domains with *ε*_*b*_ ≤ *ε* can be photoexcited); hence, the photon flux for each photon energy can be distinguished based on the extent of the photoexcited domain. When the bandgap gradient is uniform, a constant *ε* variation can lead to a fixed change in the photoexcited domain. Thus, this part of the device may present a linear response to *ε* and is expected to output a current (*I*_*2*_) that reflects the $\bar \varepsilon _\varphi$ of spectra. Combining the two output currents (*I*_*1*_ and *I*_*2*_), the spectral information may be projected into statistical spectral indices $\bar \varepsilon _\varphi$ and *φ*. On the other hand, the human visual system only perceives red, green, and blue and projects these colours in terms of hue (Fig. 1d). Labelling the flux of red, green, and blue photons as R, G, and B, respectively, the hue is defined as:

1 $$h = \frac{1}{{2\pi }}{\mathrm{atan2}}\left( {\sqrt {3}\left({G - B}\right), R - G - B}\right)$$

Figure 1e shows that the hue and $\bar \varepsilon _\varphi$ of a spectrum composed of R, G, and B are strikingly similar from the red to blue region. Therefore, except for the unphysical red–purple–blue quadrant, it is expected that a device measuring $\bar \varepsilon _\varphi$ will yield a fairly good approximation for the hue of light and thus achieve colour perception.

### Bandgap-gradient semiconductors and devices

**Fig. 2:**
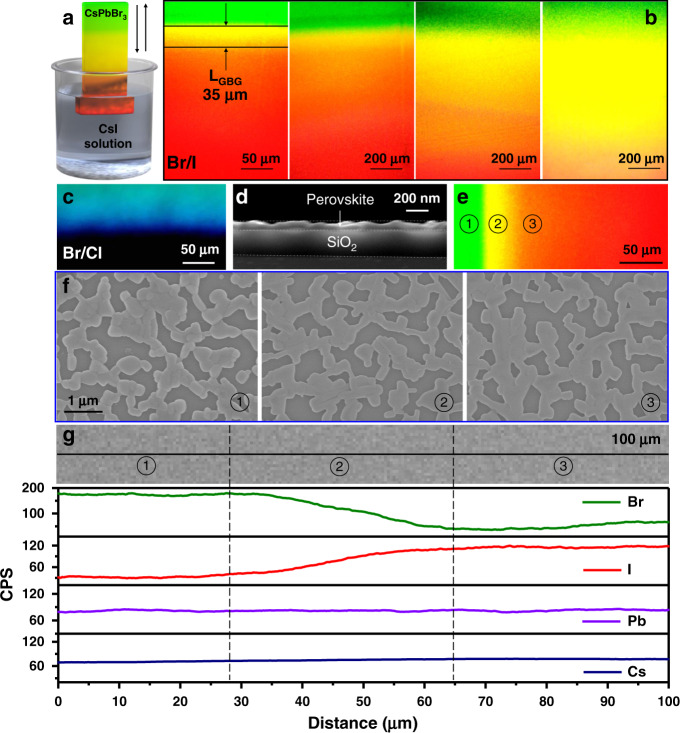
Fabrication and characterization of rainbow perovskite films. **a** Scheme of the halide-exchanging method via dipping. Fluorescence microscopy photo(s) of **b** Br/I GBG films with different *L*_*GBG*_ (the dipping speeds from left to right are 1, 2, 3, and 5 mm/s, respectively, and the concentration of the CsI solution is 16 g/L) and **c** a Br/Cl GBG film (the dipping speed is 1 mm/s and the concentration of the CsCl solution is 16 g/L). **d** Cross-sectional SEM image of a Br/I GBG film. **e** Marked positions on the Br/I GBG film. **f** SEM images and **g** linear scan of the elemental distributions around the different marked positions on the Br/I GBG film.

Semiconductors with GBGs can be achieved with compositionally graded alloys or compounds. All-inorganic halide perovskites (CsPbX_3_) exhibit tunable direct bandgaps over the whole visible range (1.7–3.1 eV), outstanding optoelectronic properties and solution processability^2,3,6,21–27^. Compared to organic–inorganic hybrid perovskites (e.g., MAPbX_3_ and FAPbX_3_), all-inorganic perovskites exhibit better robustness and environmental stability^28^. Although the cubic perovskite phase of CsPbI_3_ is relatively unstable, CsPbI_3_ can remain in the cubic perovskite phase at room temperature for a period of time, while exhibiting excellent optoelectronic properties^29^. Furthermore, CsPbI_3_ perovskites obtained from a halide exchanging CsPbBr_3_ present better phase stability than directly synthesized perovskites^30^. Thus, we use CsPbX_3_ perovskites to demonstrate this proof-of-concept. A halogen-exchange method via dipping is developed to obtain perovskite films with a continuous halide-composition gradient as shown in Fig. 2a. Figure 2b shows the fluorescence microscopy photos of a series of CsPbBr_3_ films on SiO_2_ exchanged with CsI solutions after the dipping process. According to the pictures, the photoluminescent hue of the exchanged films is spectrally continuous, changing from green to red under UV light (namely, rainbow perovskites). Similarly, the hue of CsPbBr_3_ films exchanged with CsCl solutions varies gradually from green to dark blue (Fig. 2c). Since the photoluminescence (PL) colours of perovskites depend mainly on their bandgaps^3,21,31^, these PL measurements are strong evidence that these rainbow perovskite films present continuously varying bandgaps. In this dipped exchange process, the bottom sections of the CsPbBr_3_ films undergo halide exchange with the CsCl or CsI solution for a longer duration than the top sections, spontaneously forming perovskite films with a gradient halide composition, i.e., GBGs. Interestingly, the length of the GBG range (*L*_*GBG*_) can be increased by increasing the dipping speed. From left to right in Fig. 2b, the dipping speed increases from 1 to 5 mm/s, and thus, the corresponding *L*_*GBG*_ increases from 35 μm to several millimetres. The micromorphologies of the rainbow perovskites are observed by scanning electron microscopy (SEM). Figure 2d presents the cross-section SEM image of the rainbow perovskite, showing a film thickness of ~100 nm. This thin film ensures uniform halogen exchange between the surface and the bulk of the film^31^. At different positions on the rainbow perovskite (marked in Fig. 2e), the SEM images present similar morphologies of connected grains with rather high coverage (Fig. 2f and Supplementary Fig. S1). These results reveal that the effect of the dipping process on the perovskite micromorphology is limited. The energy dispersive spectroscopy (EDS) linear and mapping scans of elemental distributions were also carried out on the GBG films around the marked positions (Fig. 2g and Supplementary Fig. S1). At position ①, clear distributions of Br, Cs and Pb can be observed, while the presence of I is negligible. From position ① to ③, the concentration of Br continuously decreases and that of I continues to increase, while the amounts of Cs and Pb remain constant. No obvious halide-phase separation was found according to the EDS mapping images. This result indicates successful halogen exchange during the dipping process.

When the exchanging solutions are diluted or the GBG perovskites are fabricated on polymer films, the *L*_*GBG*_ can be further increased. According to the UV–visible absorption, crystalline behaviour and PL spectrum (Supplementary Figs. S2 and S3), these rainbow perovskite films with a large *L*_*GBG*_ also display more spread-out bandgap gradients. The XRD spectrum around position “Br” clearly shows characteristic diffraction peaks at 15.2 and 30.7°, which can be attributed to the (100) and (200) orientations of the cubic CsPbBr_3_ crystal. After exchange with Cl or I ions, the XRD peaks exhibit obvious shifts to larger or smaller 2*θ* values (Supplementary Fig. S2c). This is ascribed to the changed lattice constants incurred by the exchange between the Br^−^ of CsPbBr_3_ and the Cl^−^ or I^−^ in the solution^31,32^. In brief, the above results indicate that the CsPbX_3_ film mainly retains a cubic crystal phase and direct bandgap after undergoing the halogen-exchange process via dipping. The weak diffraction peaks at ~27° for the Br/I perovskite and at ~27 and 45° for the Br/Cl perovskite can be assigned to a few orthorhombic and non-perovskite CsPb_2_X_5_ phases that are formed during the dipping process^33^. The mechanism of the dipping exchange process is discussed in Supplementary Fig. S4. Overall, a successful fabrication of GBG semiconductor films is displayed.

To further investigate the optoelectronic characteristics of the GBG semiconductor along the direction of the bandgap gradient, multiple devices are fabricated at different locations on the rainbow perovskite film with an *L*_*GBG*_ of ~4 mm. The photoconductivity response curves under monochromatic light exhibit a sharp cutoff for wavelengths varying from 470 to 690 nm, which is consistent with the bandgap gradient (Supplementary Fig. S5). However, the devices with ion-exchanged perovskites exhibit a high resistance, which may be due to the formation of a few non-cubic-perovskite phases as mentioned above^33^. The difference in the conductivities of the GBG composites is likely to deteriorate the hue perception of the devices. Thus, an organic semiconductor, dioctylbenzothieno [2,3-b] benzothiophene (C8BTBT), is introduced to construct heterojunctions with the rainbow perovskites^34,35^, and similar conductivities are then achieved on the heterojunctions of rainbow perovskites with different bandgaps (Supplementary Figs. S6–S8). Note that we aim to demonstrate the colour-perception devices, thus, the conducting and intensity-sensing properties of the rainbow perovskites are not optimized here.

### Linear response to photon energy and device modelling

**Fig. 3:**
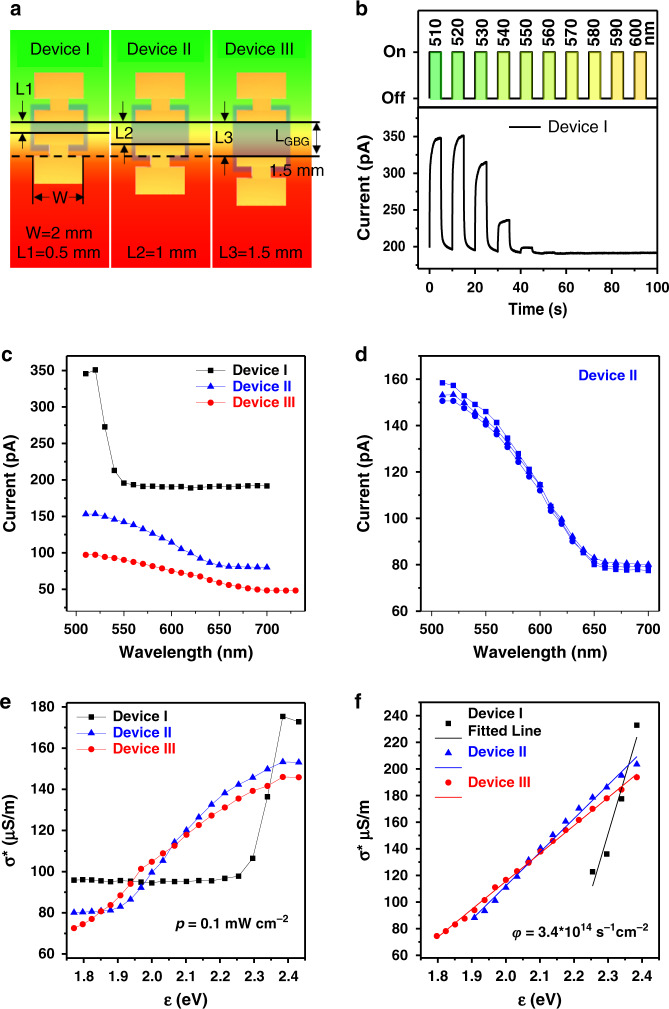
Photo-energy responsive performance of the GBG devices. **a** Schemes of parallel devices with different electrode intervals (Devices I, II, and III) using Br/I GBG films (*L*_*GBG*_ = 1.5 mm). **b** *I–t* curve of Device I under pulse lights at varying wavelengths and a step of 10 nm (5 V, 0.1 mW/cm^2^). **c** *I–λ* curves of Devices I, II, and III and **d** the sample to sample reproducibility of the *I-λ* curves (5 V, 0.1 mW/cm^2^). Plot of σ* versus *ε* for devices under a constant **e** light power density (0.1 mW/cm^2^) and **f** photo flux (3.4 × 10^14^ s^−1^ cm^−2^) together with fitted lines.

Br/I rainbow perovskite films with an *L*_*GBG*_ of ~1.5 mm are employed to fabricate the heterojunctions, and only single devices are built on the GBG films, as shown in Fig. 3a. The devices, with an electric current direction parallel to the bandgap-gradient direction (i.e., parallel devices), are named Devices I, II and III, corresponding to electrode intervals of 0.5, 1, and 1.5 mm, respectively. According to the time-dependent current curves (*I–t*) of Device I under pulsed monochromatic illumination at different wavelengths (Fig. 3b), the photocurrent decreases with increasing wavelengths from 520 to 560 nm. Figure 3c shows the current versus wavelength (*I–λ*) curves of the three devices under different monochromatic lights at a constant power density. All the devices exhibit quasi-linear current variation as a function of the light wavelengths in some regions; furthermore, the quasi-linear region widens, and the corresponding slope gradually decreases with the electrode interval. For a given *L*_*GBG*_, a large electrode interval means a wide range of varying bandgaps covered by the device. Thus, because Device III covers the whole Br/I GBG film, it exhibits a linear wavelength-responsive region from 520 to 700 nm. The linear scaling of the maximum conductivity suggests that the current differences between the three devices are mainly due to their different electrode intervals, rather than the differences in the film conductivities. An excellent sample for sample reproduction is found for the devices, as shown in Fig. 3d. The stability of the GBG device is also investigated by measuring the *I–λ* curves of the device under different ageing conditions. The device shows limited *I–λ* curve changes after many cycles of light switching (at different intensities), under an applied bias or after being stored in atmosphere for different periods of time, thereby indicating decent stability (Supplementary Fig. S9). On the other hand, the effect of the conducting current direction (parallel or orthogonal) on the wavelength-responsive performance of the device is limited (Supplementary Fig. S10). An effective conductivity (σ*) is introduced for the entire GBG device, and σ* is derived as a function of photon energy (*ε*) under a constant power density, as shown in Fig. 3e. Then, physical models for the devices are proposed (see Supplementary Information)^36^, which eventually gives:

2 $$\sigma ^ \ast = \frac{{\xi \sigma _d}}{{kl}}\left( {\varepsilon - \varepsilon _0} \right)\varphi + \sigma _d$$

where *ξ* is an experimental constant related to the dark carrier surface density and free-carrier lifetime, *σ*_*d*_ is the dark conductivity, *k* is the bandgap gradient, and *l* and *ε*_*0*_ are the length and the smallest bandgap of the GBG film employed by the device, respectively. This equation means that for a constant photon flux *φ*, the devices should show an ideal linear response to *ε* (*I*~*ε*). In Fig. 3f, the σ* *−* *ε* dataset with constant illumination power *p* in the quasi-linear region is recasted with constant *φ* (*φ* = *p*/*ε*). The curves can be well fitted to a straight line when *φ* is constant, which supports physical modelling. According to the model, the linear photon energy response also benefits from the heterojunction device structure. The photogenerated charge carriers from the rainbow perovskite transfer into the uniformly conductive C8BTBT layer and then conducts, thereby avoiding the influence of the conductivity difference in the rainbow perovskites (Supplementary Figs. S11 and S12 and Tables S1 and S2).

Then, the photogenerated current (*I*_*ph*_, equal to light current minus dark current) of the device under the given voltages can be expressed as:

3 $$I_{ph} \propto \left( {\varepsilon - \varepsilon _0} \right)\varphi = p - \varepsilon _0\varphi$$

which suggests that the photocurrent is not solely determined by *p* despite the fact that it combines *ε* and *φ* (*I* *≠* f (*p*)). Thus, the device can distinguish different light hues even with the same *p*, and the *ε* and *φ* of the light can be obtained by combining the device with any conventional photodetector. For non-monochromatic light, the total photocurrent in devices is the sum of the contribution of each monochromatic light source:

4 $$I_{ph} = \mathop {\sum}\limits_i {I_{phi}} \propto \mathop {\sum}\limits_i {\left( {\varepsilon _i - \varepsilon _0} \right)\varphi _i = \left( {\overline {\varepsilon _\varphi } - \varepsilon _0} \right)\varphi }$$

which directly indicates the statistical spectral indices (here *φ* is the total photon flux of the spectra). This means that the GBG device is able to project spectra and directly provide average photon energies, i.e., the hue. A GBG device covering the full visible spectrum can be further obtained by dipping the perovskite film half in the CsCl solution and half in the CsI solution, with optimized dip-processing parameters to ensure a good match of the bandgap gradients for both the Br/Cl and Br/I parts.

### Spectrum projection and colour perception

**Fig. 4:**
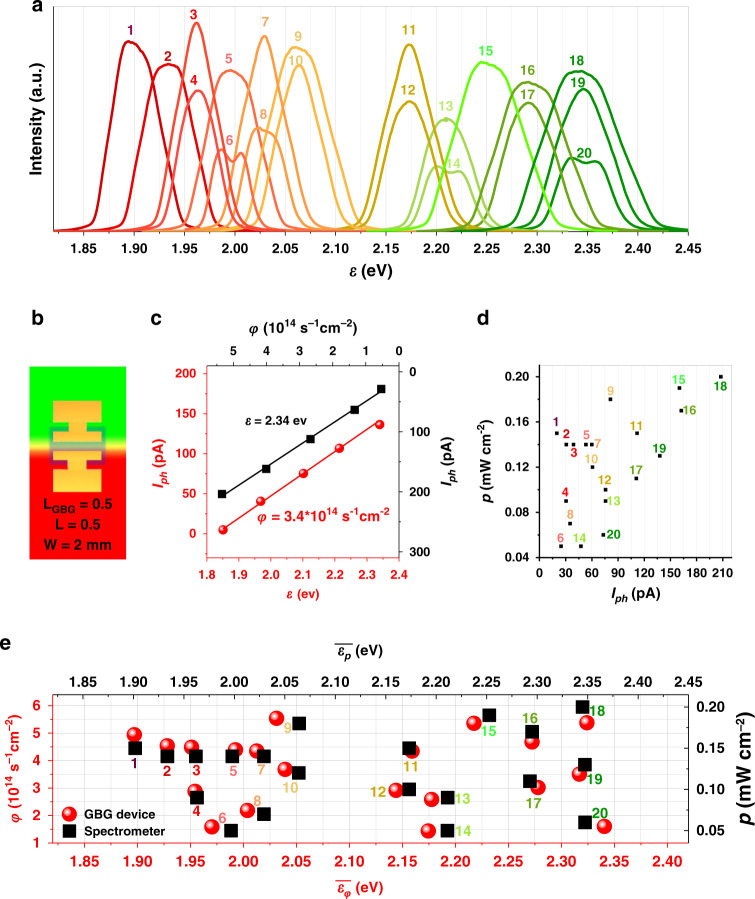
Spectrum projection using the GBG device. **a** Spectral diagrams of 20 different radiations measured by a spectrometer, and the intensities of the curves are normalized according to the measurements of a light power density metre. **b** Scheme of the GBG device that is used. **c** Calibration of the GBG device (5 V). **d** Photogenerated currents (*I*_ph_) of the GBG device for the 20 incident radiations (5 V). **e** The $\bar \varepsilon _\varphi$ value of the radiation measured by the GBG device compared with the $\bar \varepsilon _p$ value obtained from the spectrometer.

Figure 4a shows spectral curves of 20 radiations measured by a commercial spectrometer, distributed in the range of 520–670 nm (1.85–2.38 eV). Since the spectrometer can only provide relative and imprecise light intensities, the spectral curves are normalized according to the more accurate power densities measured by a light power density metre. A GBG heterojunction device containing a Br/I rainbow perovskite film with an *L*_*GBG*_ of ~0.5 mm is used here (Fig. 4b). The calibration of the GBG device is first carried out by reading *I*_*ph*_ under different monochromatic lights with varying *ε* or *φ* (Fig. 4c). Both the *I*_*ph* _− *ε* and *I*_*ph*_ − *φ* curves appear to be linear in our experimental *ε* and *φ* regions, and the calibration parameters for the device are obtained accordingly (see the Supplementary Information). The device is then employed to measure the 20 radiations and provide the corresponding *I*_*ph*_ presented in Fig. 4d, together with the power densities. The different radiations with the same power densities (e.g., Radiation 1 and 11 with *p* = 0.15 mW cm^−2^, Radiation 2, 3, 5 and 7 with *p* = 0.14 mW cm^−2^, Radiation 4 and 13 with *p* = 0.09 mW cm^−2^, and Radiation 6 and 14 with *p* = 0.05 mW cm^−2^) clearly exhibit different *I*_*ph*_ values, indicating that the radiations that are undistinguishable with commercial light power density metres can be identified by our device. Furthermore, the statistical spectral information of the radiation, including $\bar \varepsilon _\varphi$ and *φ*, can be obtained from *I*_*ph*_ and *p*, as shown in Fig. 4e. For comparison, the average photon energy weighted by the power density ($\bar \varepsilon _p$) obtained from a spectrometer is also shown. The values of $\bar \varepsilon _\varphi$ and $\bar \varepsilon _p$ for each radiation are close to each other, and both are located in the mid-region of the corresponding spectrum (see the Supplementary Information). This result reveals that the output *I*_*ph*_ of the GBG device reliably indicates the $\bar \varepsilon _\varphi$ value of the radiation. The results of $\bar \varepsilon _\varphi$ and $\bar \varepsilon _p$ show slight deviations for some spectra, which can be ascribed to (1) the two average photon energies ($\bar \varepsilon _\varphi$ and $\bar \varepsilon _p$) are weighted by different parameters, i.e., the former (obtained with the GBG device) is weighted by photon flux, and the latter (obtained with a spectrometer) is weighted by power density and (2) the photosensitivity and spectral resolution of the GBG device can be further improved.

**Fig. 5:**
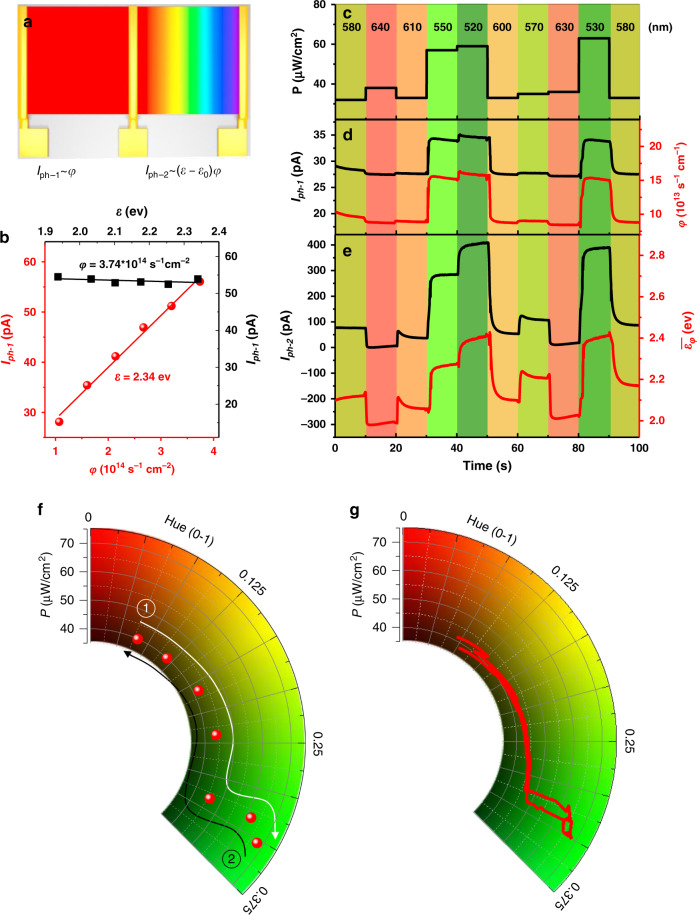
Online colour perception using a single device. **a** Scheme of the device consisting of two parts and the output of the two photogenerated currents (*I*_*ph-1*_ and *I*_*ph-2*_). **b** Calibration of *I*_*ph-1*_. **c** Real-time changes in light with randomly varying colours and intensities. **d** *I*_*ph-1*_ variations and the accordingly calculated *φ*–*t* curves and **e** *I*_*ph-2*_ variations along with the obtained $\bar \varepsilon _\varphi$–*t* curves that are responsive to the changing lights (5 V). **f** Track of changing lights with monotonically increasing (decreasing) hues and intensities, and **g** the measured light-varying track in the hue coordinate system.

Then, a device consisting of two parts that employ the GBG film and the SBG CsPbI_3_ film as photoactive layers is fabricated with a rainbow perovskite (Fig. 5a). Similar to conventional direct-bandgap semiconductive photodetectors, the SBG part exhibits a linear response to *φ* and very limited current changes as a function of *ε* in our experimental regions (Fig. 5b). The device can thus project the spectrum and perceive colours by combining the two photogenerated currents of the SBG and GBG parts (*I*_*ph-1*_ and *I*_*ph-2*_). Figure 5c shows time-dependent lights with randomly varying colours and intensities (i.e., radiations with changing spectra in real time), which are generated by a xenon lamp filtered with two tunable grating monochromators. Figure 5d exhibits *I*_*ph-1*_ variations from the SBG part of the device under the changing light. Since *I*_*ph-1*_ is only sensitive to *φ*, *φ*–*t* curves are calculated accordingly. Moreover, the GBG part of the device under the lights changing in real time provides online *I*_*ph-2*_–*t* curves. The $\bar \varepsilon _\varphi$–*t* curves, namely, the online colour-varying information, can be simultaneously obtained from the known *φ* and the *I*_*ph-2*_–*t* curves (Fig. 5e). In addition, the track of changing lights with monotonically increasing (decreasing) photon energies is shown in a hue coordinate system, in which the scale of the radial axis represents light intensity. Figure 5f exhibits the pre-set light-changing situations, and Fig. 5g shows the measured results from the device. In all cases (Fig. 5c–g), the results obtained from the device are similar to the expected results, indicating reliable measurements. To the best of our knowledge, this is the first study to show online colour perception on a single device.

Note that humans perceive mixed red (~1.8 eV) and blue (~2.8 eV) colours as purple; hence, our device may be unable to distinguish between the “mixed purple” and yellow (~2.3 eV) signals since they can have the same average photon energy. An extra electrode deposited in the middle of the green region on the GBG device can overcome this issue by providing average photon energies of the red–green and green–blue regions, respectively. It is also worthwhile to compare our device with the conventional pixel sensor in charge-coupled device (CCD) cameras, which consists of several photodetectors integrated with red, green, and blue filters. The CCD pixel device collects spectral information in three different wavelength ranges (mimicking human eyes) and then provides the hue by combining the R, G, and B values. Thus, the obtained hue highly depends on the optical properties of the R, G, and B filters. In contrast, our device discriminatively collects spectral information in many (theoretically infinite) different wavelength ranges and provides an average photon energy. The obtained average photon energy is an intrinsic parameter of spectra, which can be further transformed into a hue. Moreover, the device configurations of the two methods are completely different. The CCD pixel sensor requires at least three photodetectors integrated with three different optical (RGB) filters, whereas our devices only require a bandgap-gradient semiconductor. The advantages of colour sensors utilizing our method include a high spectrum resolution, low cost, simple patterning and scaling down, simple fabrication on flexible substrates, and simple customization, while eliminating the need to find good filters.

### Demonstration of device flexibility and chemical/bio-sensing

**Fig. 6:**
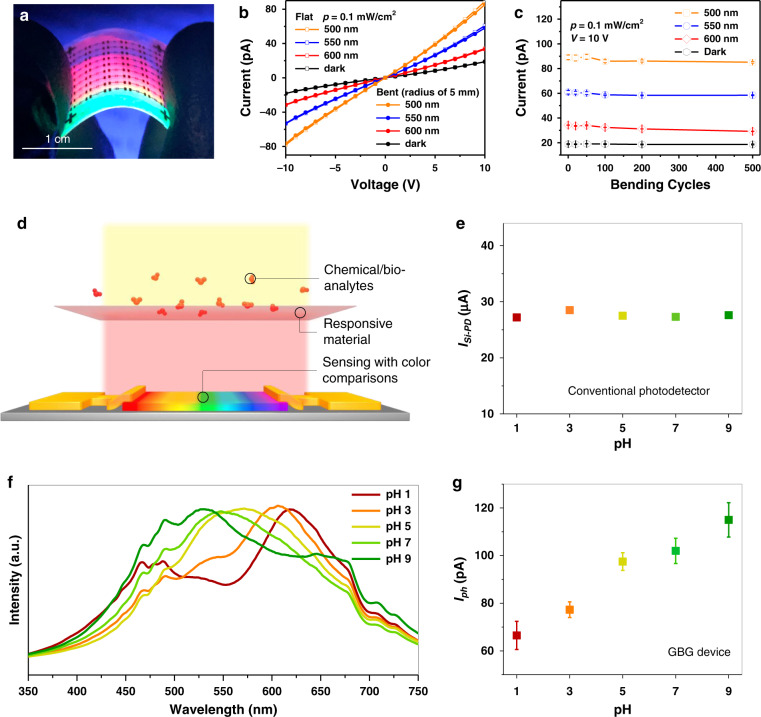
Demonstration of device flexibility and chemical/bio-sensing. **a** An image of the flexible devices under UV illumination. **b** *I*–*V* curves of the flat and bent flexible device in the dark and at different wavelengths of light, respectively (bent radius is 5 mm). **c** Variations in the currents of the flexible device as a function of bending cycles at different wavelengths of light. **d** Scheme of applying the GBG device in chemical/bio-sensors, in which the chemical/bio-analytes (e.g., proton concentrations in solutions) result in colour changes of stimuli-responsive materials (e.g., pH testing papers), and the GBG device senses the stimuli with colour comparisons. Measurements of the colour responses to pH testing papers in solutions with different proton concentrations (i.e., pH values of 1, 3, 5, 7, and 9) with **e** a silicon photodiode, **f** a spectrometer, and **g** our GBG device (error bars in **c** and **g** are obtained from 5 measurements).

The mechanical flexibility is also demonstrated by fabricating GBG devices on polymer substrates (Supplementary Fig. S13). Figure 6a shows an image of the device array under UV illumination, showing spectrally gradient PL colours and mechanical flexibility. The flexible devices also exhibit *I*–*V* curves responsive to light wavelengths, and the *I*–*V* curves remain the same when the devices are in a bent state (Fig. 6b). The currents of the flexible devices under light at various wavelengths exhibit only slight changes after undergoing hundreds of bending cycles (Fig. 6c), thereby indicating decent flexibility. The flexibility may further extend the applicability of the device^37,38^.

Finally, chemical/bio-sensing by applying the colour-perception device is demonstrated. The colorimetric chemical/bio-assay setup is shown in Fig. 6d: chemical/bio-analytes will modify the colour of the sensing material, and the GBG device will convert the colour change to an electrical signal. For this simple proof of concept, we use a pH testing paper that turns from red to green for pH values from 1 to 9. When using a single silicon photodiode output current *I*_*Si-PD*_, such colour differences are not detectable, as shown in Fig. 6e. A spectrometer is then employed to measure the spectra of the pH testing papers under the same illumination, which can distinguish the testing papers with different colours by presenting different peak positions of the spectral curves (Fig. 6f). However, this method not only requires bulky equipment but also generates spectral information that is redundant for sensing applications. The response of the hue sensor is shown in Fig. 6g. Our device can clearly distinguish between the various pH values. Therefore, the hue sensor effectively achieves colorimetric chemical/bio-sensing with only a single device and by generating only one or two current signals.

## Discussion

In conclusion, we proposed a concept of spectrum projection and colour perception on a single cell inspired by human visual perception and manufactured such a device by developing bandgap-gradient rainbow perovskites. The GBG part of the device can project the spectrum into the average photon energy and photon flux, while the SBG part can only indicate the photon flux of the spectrum. Thus, the intensity and colour (hue) of the spectrum can be obtained from a single device by combining the two output signals, which are in good agreement with those obtained from a commercial photodetector and spectrometer. Online colour perception in a real-time light-varying environment was demonstrated by a single device. Furthermore, flexible devices were obtained by depositing rainbow perovskites on a polymer substrate. Simple chemical/bio-sensing was further shown by applying the device in a colour comparison experiment.

We think that bandgap-gradient structures with high degrees of control can be achieved by other fabrication technologies with processing parameters that can produce a gradient, i.e., the technology of inkjet printing with gradients available in the substrate temperature or ink concentration. Such localized patterns of bandgap gradients possess major potential applications, including multicolour LEDs and complex designs of colour sensors. In addition to the rainbow perovskite, the device can also be obtained by using various compositionally graded semiconductors (such as Si_1−*x*_Ge_*x*_ (0.7–1.1 eV), V_*x*_Zn_*y*_O (0.6–3.3 eV), ZnO_*x*_N_*y*_ (1.0–3.3 eV), In_*x*_Ga_1−*x*_N (0.7–3.4 eV), and CdS_*x*_Se_1−*x*_ (1.7–2.4 eV)), multiple device configurations (including semiconductor films with vertical bandgap-gradient distributions, conducting films blended with bandgap-gradient semiconductor nanoparticles, and semiconductor nanowires with axial GBGs) and different fabrication technologies^14,15,39–42^, which can lead to a variety of device characteristics. For instance, arrays of devices with high photon energy responsivity can be obtained by using conducting films blended with bandgap-gradient CsPbX_3_ quantum dots^43^. Atomic layer-deposited oxide and nitride semiconductor thin films with vertical bandgap-gradient distributions can scale down devices to the nanometre scale and provide devices with compatibility to Complementary Metal Oxide Semiconductor technology^39,42^. This device can be used in colour-sensing pixels, which may be more simplified than existing devices containing several photodetectors and optical filters. Multifunctional sensors can be produced by combining devices with stimuli-responsive materials to detect physical/chemical/bio-stimuli through a comparison of colours/spectra^44^. Therefore, this work provides a new category of optoelectronic devices that are capable of spectrum projection and hue perception, thereby opening up a range of colourful applications.

## Materials and methods

### Preparation and characterization of rainbow perovskite films

First, pure CsPbBr_3_ perovskite films were prepared by spin-coating the precursor solution (2 mmol of CsBr and 2 mmol of PbBr_2_ in 5 ml of dimethyl sulfoxide)) at 3000 rpm for 1 min on SiO_2_- or polylactide (PLA)-coated Si wafers, followed by drying at 70 °C for 30 min in a N_2_ atmosphere. The PLA films were fabricated by spin-coating PLA solution (Natureworks Co., Ltd., Unitied States, 4032D, 50 g/L in chloroform) onto the wafer at 2000 rpm for 30 s, followed by heating at 60 °C for 2 h. Then, the rainbow perovskite films were obtained by dipping the CsPbBr_3_ films (on a wafer or PLA) in the CsCl/CsI methanol solutions at a concentration of 4, 8 or 16 g/L. The dipping speeds of the CsPbBr_3_ films in the solution were 1, 2, 3, or 5 mm/s, and the pulling speed was 5 mm/s for all GBG films (Dip Coater, SYDC-100, SANYAN Co., Ltd., China). The fluorescence microscopy photos were taken by using a fluorescence microscope (LW300LFT-LED, Cewei Guangdian Co., Ltd., China) with an excitation light at 365 nm. The images of the rainbow perovskites were taken by a digital camera under UV lamp illumination with a wavelength at 365 nm. SEM images were obtained from a field-emission electron microscope (Zeiss SIGAMA HD, Germany) operated at 5 kV. EDS images were taken by a Zeiss Gemini SEM500 field-emission scanning electron microscope with an Aztec X-Max Extreme EDS detector (Oxford Co., Ltd., Britain). UV–vis absorption spectra for the GBG films on quartz substrates were measured on a Lambda 750 UV–visible spectrophotometer. PL spectra were recorded by an F-320 spectrophotometer (Gangdong Sci.&Tech. Co., Ltd., China). X-ray diffraction (XRD) was carried out on a Bruker (Unitied States) Advance D8 X-ray Diffractometer with a Cu Kα radiation source (1.54 Å) at 40 kV. The rainbow perovskites with rather large *L*_*GBG*_ values were cut into small pieces for the UV–vis, PL and XRD characterizations at different positions.

### Fabrication of the devices

The devices in Supplementary Fig. S5a were fabricated by using Br/Cl or Br/I rainbow perovskites on a wafer with an ~4 mm *L*_*GBG*_ as photosensing layers. Au electrodes (70 nm) were thermally evaporated onto the GBG films through a shadow mask under a vacuum of 8 × 10^−4^ Pa. The width (*W*) and length (*L*) of the device was 500 and 100 µm, respectively. The orthogonal heterojunction devices (Supplementary Fig. S6a) were fabricated by thermally evaporating a 150 nm C8BTBT film on top of the Br/I GBG film with an ~2 mm *L*_*GBG*_ using a shadow mask, followed by deposition on the electrodes as mentioned above. Similarly, the parallel heterojunction devices shown in Fig. 3a were obtained by employing Br/I GBG films on a wafer with an ~1.5 mm *L*_*GBG*_ and electrode intervals of 0.5, 1, and 1.5 mm. Other parallel and heterojunction devices were obtained by using the same method. The flexible heterojunction devices shown in Fig. 6a were prepared by depositing the Br/I GBG film on PLA film and PET substrate, followed by the evaporation of the C8BTBT and Au electrodes (*W* = 500 µm and *L* = 100 µm).

### Characterization of the devices

All electrical characteristics were measured on a Cascade (Unitied States) Summit 11000M probe station by using an Agilent (Unitied States) B1500A semiconductor device analyser in air at room temperature. The monochromatic and broadband lights were generated by either a 300 W xenon lamp filtered with two tunable grating monochromators (Omno 330150, NBeT Co., Ltd., China) or LEDs and bandpass filters in a range of wavelengths (Thorlabs Co., Ltd., Unitied States). All monochromatic lights were fixed with a full-width at half maximum of approximately 10 ± 2 nm. The power densities of the radiation were measured using a standard photodiode power density metre with the input of light (Thorlabs S130VC). The curves in Fig. 4a were obtained by employing an F-320 spectrophotometer (Gangdong Sci.&Tech. Co., Ltd., China), and the intensities of the curves were further normalized according to the power densities measured by a Thorlabs S130VC instrument, that is, the integral area of each curve was normalized to be proportional to the measured power density of the corresponding radiation. The performance of the flexible devices in the bent state was characterized while the devices were tightly bent around a cylinder with a radius of 5 mm. The pH sensing experiments were carried out by testing the GBG device under an Omno 330150 light source filtered with differently coloured pH testing papers.

## Supplementary information

Supplementary information

## Data Availability

The data that support the conclusions within this paper and the other findings of this study are available from the corresponding authors upon reasonable request.
