# Supplementary material for: Spectrum projection with a bandgap-gradient perovskite cell for colour perception
Source: Light Sci Appl. 2020 Sep 15;9:162. doi: 10.1038/s41377-020-00400-w (PMC7492220; doi:10.1038/s41377-020-00400-w)
Supplement: Supplementary file 1 — Supplementary information [file 41377_2020_400_MOESM1_ESM.docx]

Supplementary information for

**Spectrum projection with a bandgap-gradient perovskite cell for color perception**

*Mei-Na Zhang^1,4^, Xiaohan Wu^1,4^*, Antoine Riaud^1^, Xiao-Lin Wang^1^, Fengxian Xie^2^, Wen-Jun Liu^1^, Yongfeng Mei^3^, David Wei Zhang^1^ and Shi-Jin Ding^1^**

**Fig. S1.** a) SEM images with a small magnification, b) Br, c) I, d) Cs and e) Pb element distributions of the Br/I GBG film around different positions as marked in Fig. 2e.


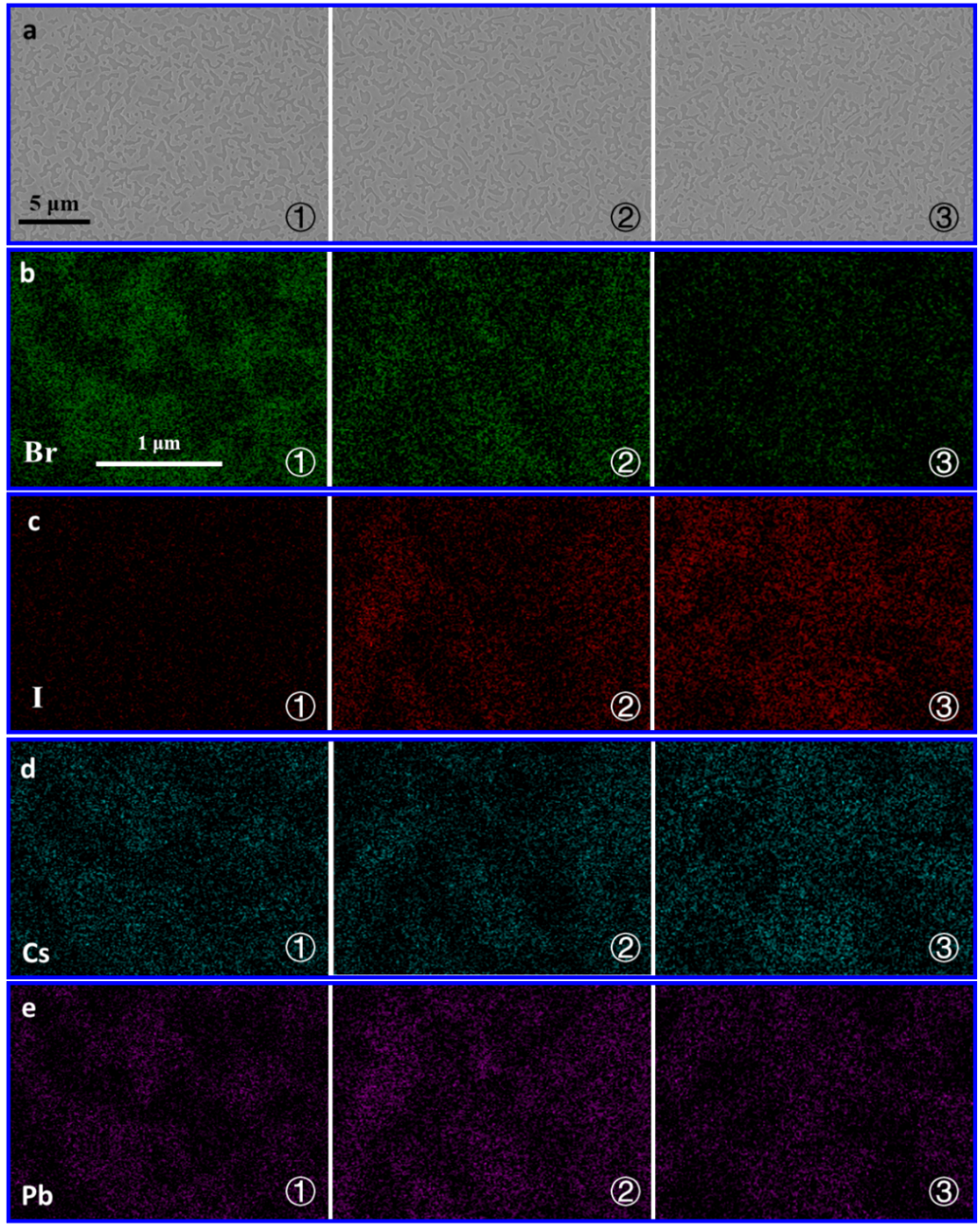


**Fig. S2.** Characterizations of the rainbow perovskites on the SiO_2_ film with large *L_GBG_*. a) Scheme of the marked positions on the Br/Cl and Br/I GBG films with a large *L_GBG_* of around 4 mm. b) UV-visible absorptions and c) XRD patterns of the GBG films associated with the marked positions.

**
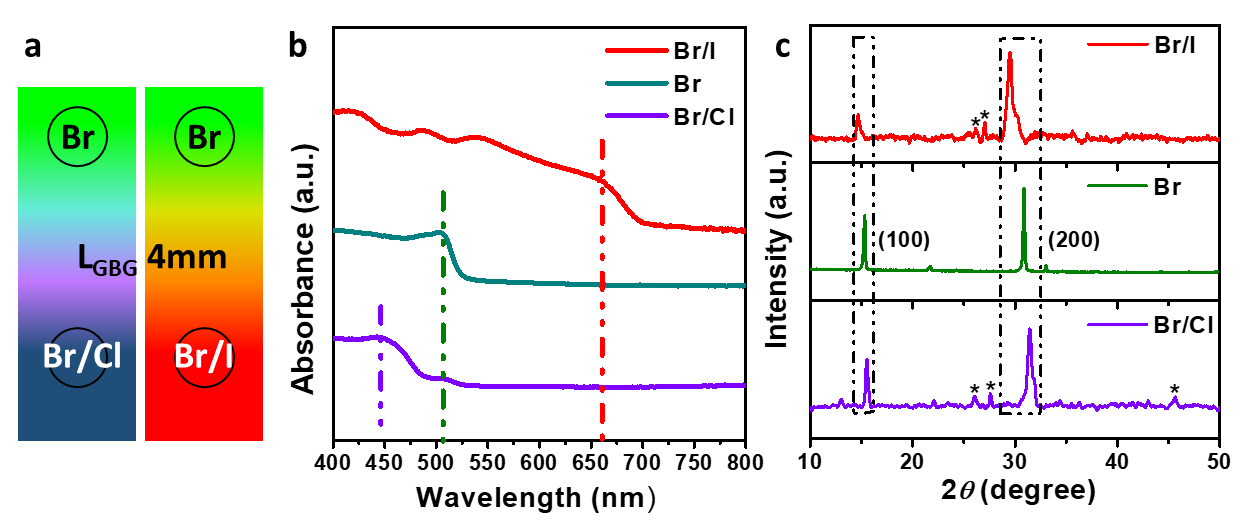
**

**Fig. S3.** Characterizations of the rainbow perovskites on the polymer film. a) An image of a CsPbBr_3_ film on the polymer film under UV illumination. Images of the b) Br/Cl and c) Br/I GBG perovskites on the polymer film under UV illumination with different *L_GBG_*. (From left to right in b) the dipping speed are 3, 3, 3, 1 and 5 mm/s, and the concentrations of the CsCl solutions are 4, 8, 16, 4, 4 g/L. From left to right in c) the dipping speed is 3, 5, 5 mm/s, and the concentration of the CsI solution is 8, 8, 4 g/L, respectively.) d) Different positions marked by numbers on the Br/Cl and Br/I GBG perovskites/polymer films. e) Fluorescence microscope photos, f) SEM images and g) PL curves of the GBG perovskites/polymer films around the different marked positions. h) A cross-section SEM image of the Br/I GBG perovskite on the polymer film.

**
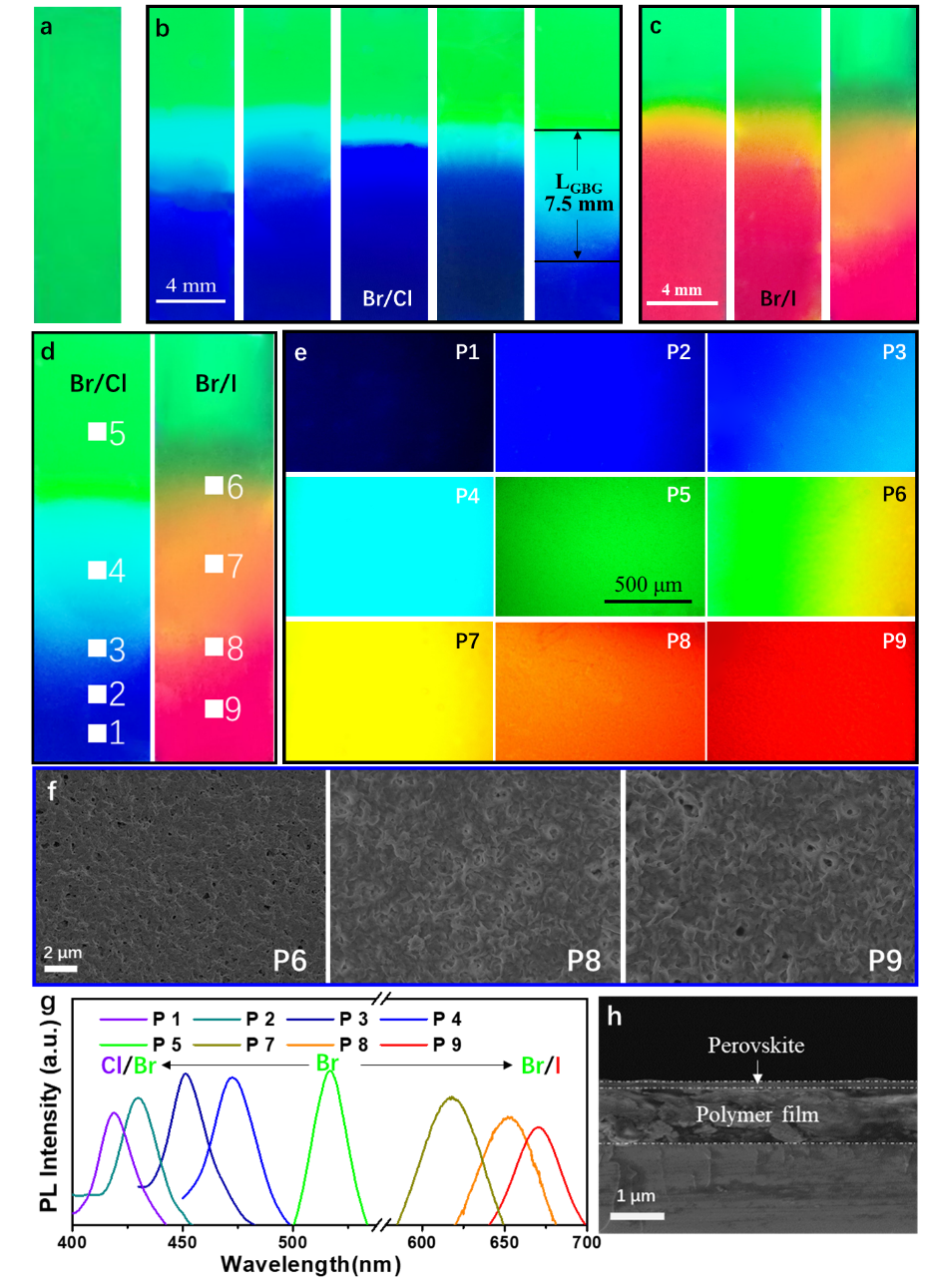
**

**1. Characterizations of the rainbow perovskites with large** ***L_GBG_***

More diluted exchanging solutions result in larger *L_GBG_*, *i.e.* Br/Cl and Br/I GBG perovskites on SiO_2_ films with *L_GBG_* of around 4 mm are fabricated using the CsCl or CsI exchanging solution with 4 g/L concentration, respectively. Fig. S2 show UV-visible absorptions and XRD patterns of the GBG films at the different marked positions (*i.e.* position “Br”, “Br/Cl” and “Br/I” as marked in Fig. S2a). The UV-visible absorption curves exhibit different cutoff points to wavelength for the perovskites around the different locations, indicating different bandgaps (Fig. S2b). Fig. S3 a-c show images of a serial of Br/Cl and Br/I GBG perovskites on polylactide (PLA) films under UV illumination, in which the *L_GBG_* of both the perovskites are much larger than those on SiO_2_ wafers. Note that even on the GBG films with *L_GBG_* close to one centimeter, the variation of the PL colors (bandgaps) is still continuous (Fig. S3d and e). Overall, the *L_GBG_* can be tuned form micrometers to centimeters by changing the dipping speed, the exchanging solution concentration and the variety of the bottom film. The micromorphologies of the GBG perovskites on PLA films are observed by SEM. Around different positions on the GBG perovskites (marked in Fig. S3d), all the SEM images present similar morphologies with relatively small crystal and full coverage (Fig. S3f), revealing a limited effect of the dipping process on their micromorphologies. The PL curves exhibit different peak positions for the perovskites around the different locations, which means that these perovskites possess different bandgaps (Fig. S3g). The CsPbBr_3_ films undergone halide exchange with the CsCl or CsI solution exhibit slightly weaker PL intensities as compared with that of the initial CsPbBr_3_ film, roughly indicating a slightly lower PL quantum yield. The results are consistent with the XRD spectra of the GBG perovskites, in which few non-cubic perovskite phases are found after the dipping halogen-exchanging process. The thickness of the perovskites on the polymer film is also around 100 nm (Fig. S3h). which avoids uneven halogen-exchanging between the surface and the bulk of the perovskites.

**Fig. S4.** The mechanism of the dipping halide-exchanging process.


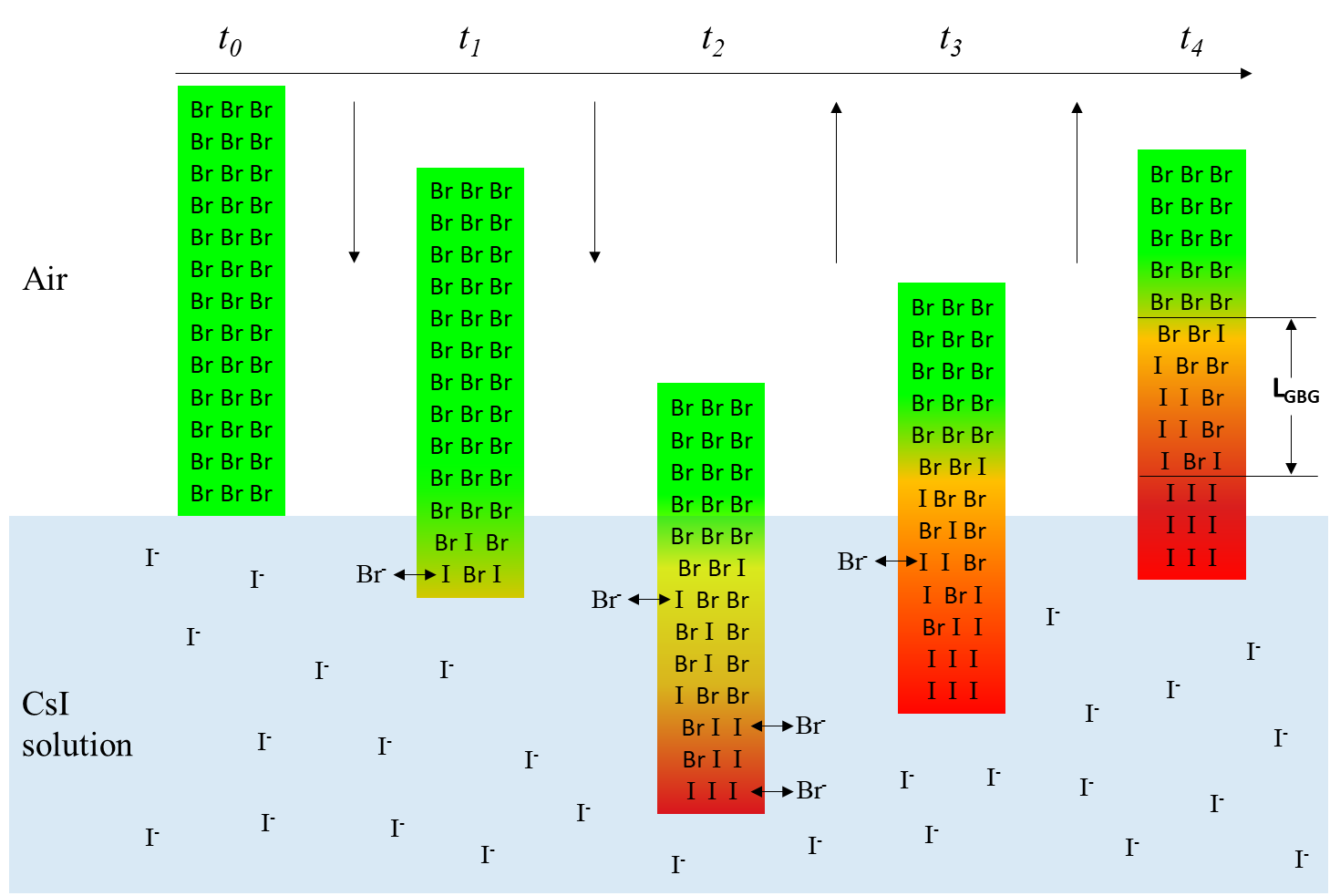


**2. A mechanism of the dipping halide-exchanging process**

Fig. S4 shows the detailed scheme of I exchanging with Br for a CsPbBr_3_ film dipping in the CsI solution. From *t_0_* to *t_2_*, the film keeps dipping down, and from *t_2_* to *t_4_*, the film remains pulling up. The film is thin enough (about 100 nm), so that we consider the halide-exchanging degree at the film thickness direction is uniform. At *t_0_*, the CsPbBr_3_ film just contacts with the surface of the CsI solution and the PL color of the whole film is still pure green. At *t_1_*, the bottom film has been dipped into the solution and slight substitution has occurred, leading to the PL color of the bottom film appears green-yellow. At *t_2_*, the film has been dipped down to the lowest position of the whole dipping process. The bottom film has experienced a rather long time exchanging while the middle part of the film has just been immersed into the solution, which leads to a rainbow PL color for the film. At *t_3_*, the film has been pulled up for a while. The middle part of the film remains a slight exchange and the bottom part possesses a large degree of halide substitution, leading to a strongly varying PL color. Finally, the film has almost been pulled out of the solution at *t_4_*, and the exchanging at the bottom film has saturated, which defines the *L_GBG_*. A higher solution concentration or a lower dipping speed would result in a fast saturation of the halide-exchanging, and thus leads to a smaller *L_GBG_*. Comparing the micromorphology of the CsPbBr_3_ film on the SiO_2_ with that on the PLA film, the former exhibits a porous polycrystal structure while the latter appears a fully covered film. The contact interface with the CsI solution of the former is relatively large, leading to a fast exchanging, namely a fast saturation. Therefore, smaller *L_GBG_* is obtained for the rainbow perovskite on the SiO_2_ than that on the PLA film.

**Fig. S5.** Optoelectronic characteristics of the devices fabricated with rainbow perovskite films. a) Schemes of multiple devices employing the Br/Cl and Br/I GBG perovskites with *L_GBG_* of around 4 mm as photoactive layers. *I-V* curves of b) Device 6 and c) Device 10 in dark and under illuminations with different wavelengths (0.15 mW cm^-2^). d) Responsive characteristics of Device 6 under pulse lights with different wavelengths (0.1 mW cm^-2^, 2 V). e) *I-t* curves of a series of devices under pulse lights with different wavelength increasing from 400 to 750 nm at a step of 10 nm per pulse (0.1 mW cm^-2^, 2 V).


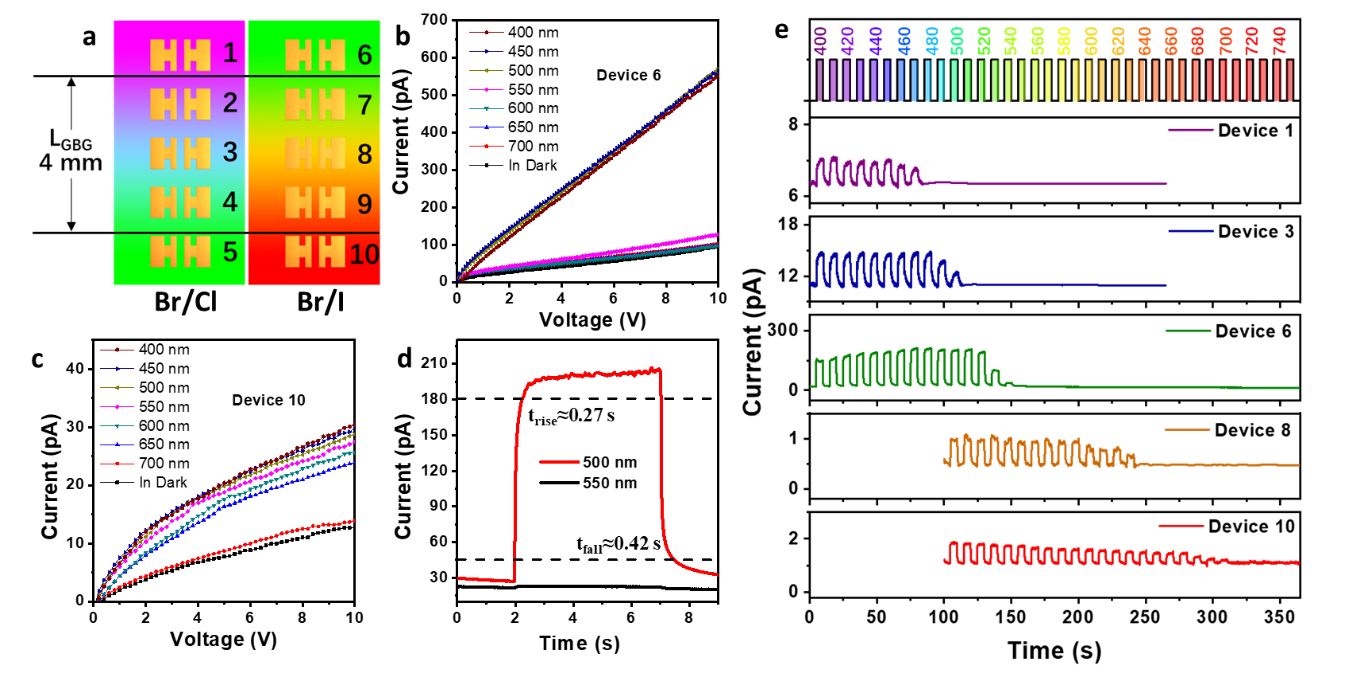


**Fig. S6.** Image and micromorphology of the rainbow perovskites/C8BTBT heterojunctions. a) A fluorescence microscope photo of the heterojunction devices with a Br/I GBG film (*L_GBG_* = 2 mm). b) Energy band alignments of the respective perovskites and C8BTBT. c), d) Top-view and e), f) cross-section SEM images of the heterojunction with different magnifications.


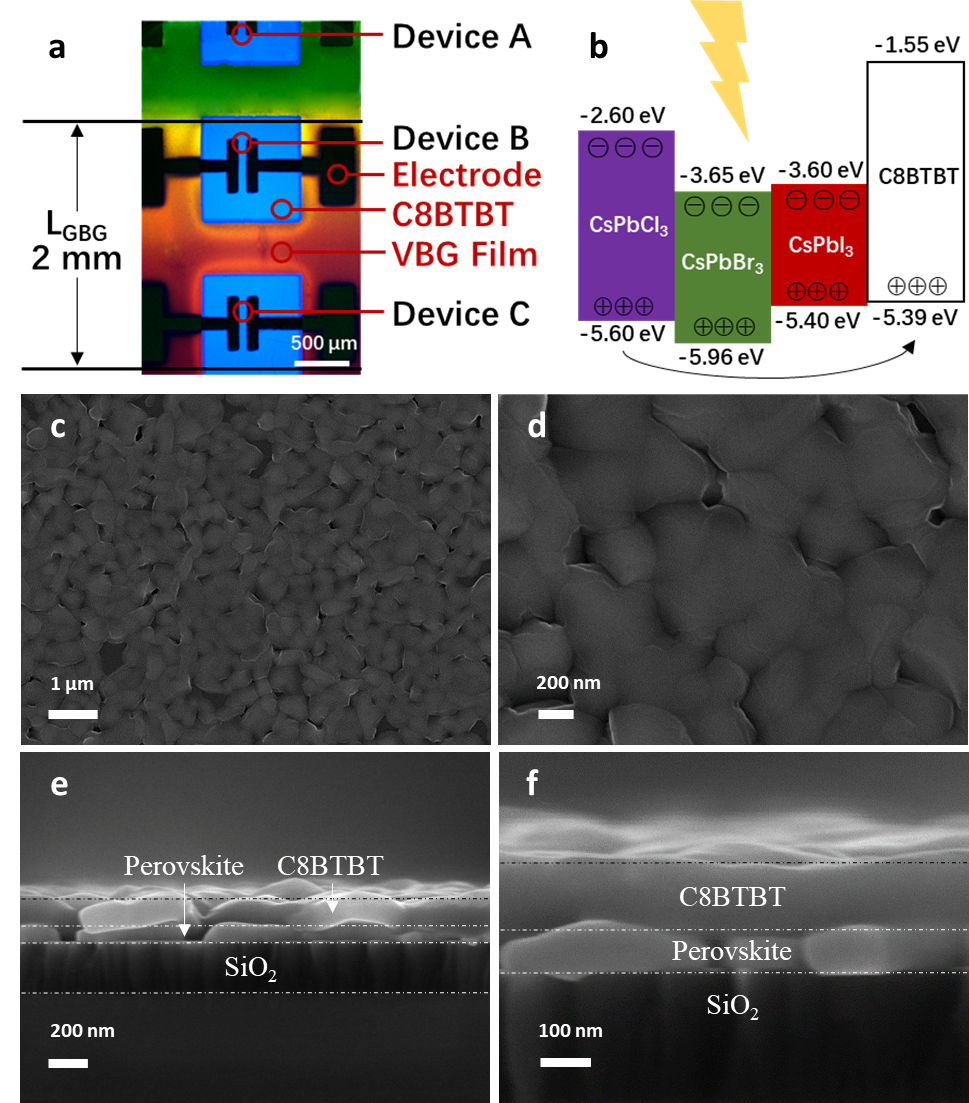


**Fig. S7.** Performance comparisons of the heterojunction and pure C8BTBT devices. *I-V* curves of Device A under lights with different a) power densities (530 nm) and b) wavelengths (0.3 mW cm^-2^). c) *I*-*t* curves of Device A under pulse lights with different power densities (530 nm, 2 V). d) UV-visible absorption of the C8BTBT film. *I*-*V* curves of the pure C8BTBT device under lights with different e) power densities (530 nm) and f) wavelengths (0.3 mW cm^-2^).

**
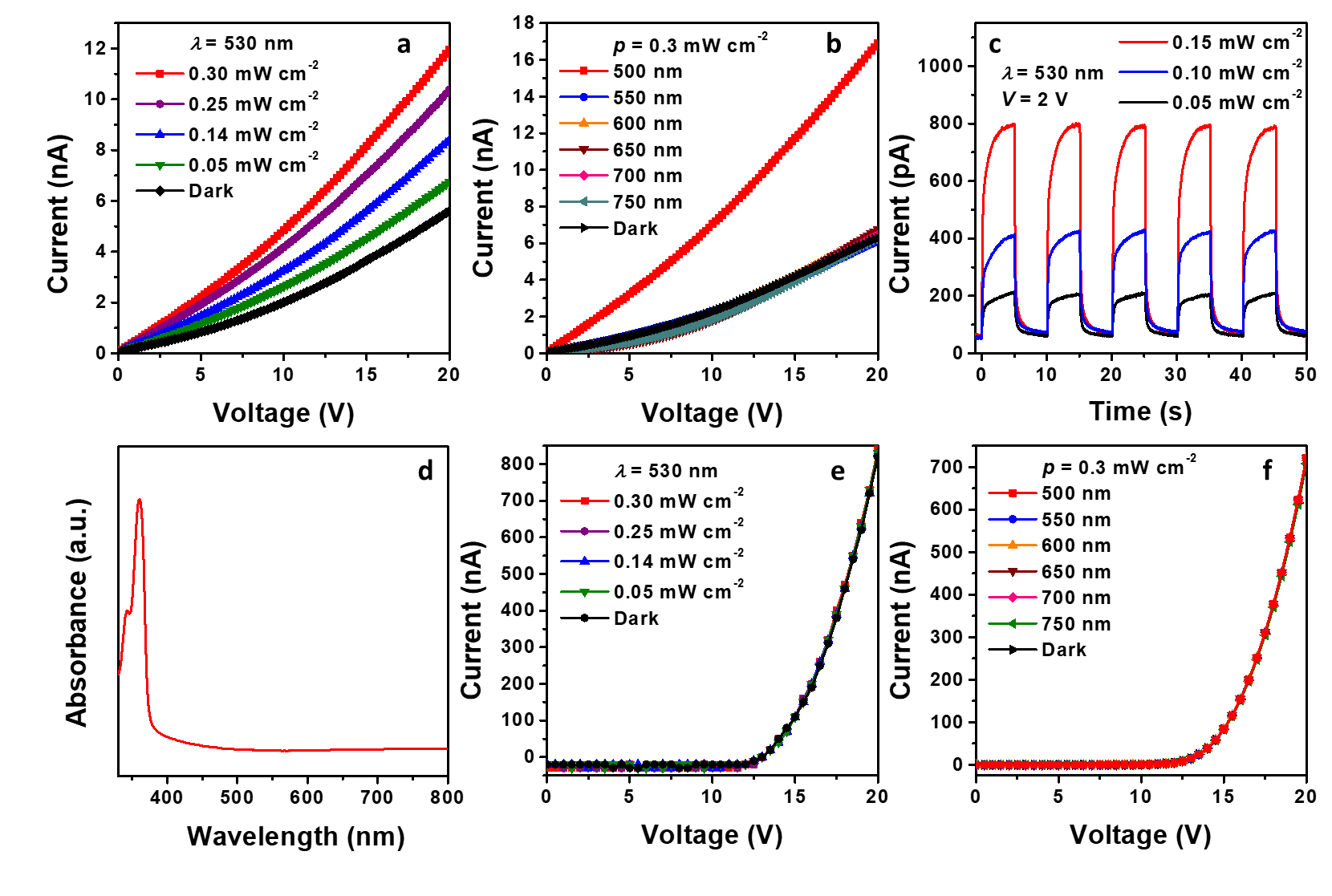
**

**Fig. S8.** Optoelectronic characteristics of the GBG heterojunction devices. *I-t* curves of the heterojunction devices under circular pulse lights with different wavelengths for a) Device A, b) Device B and c) Device C (2 V, 0.1 mW cm^-2^). d) *I-λ* curves of Device B and C (5 V, 0.1 mW cm^-2^).

**
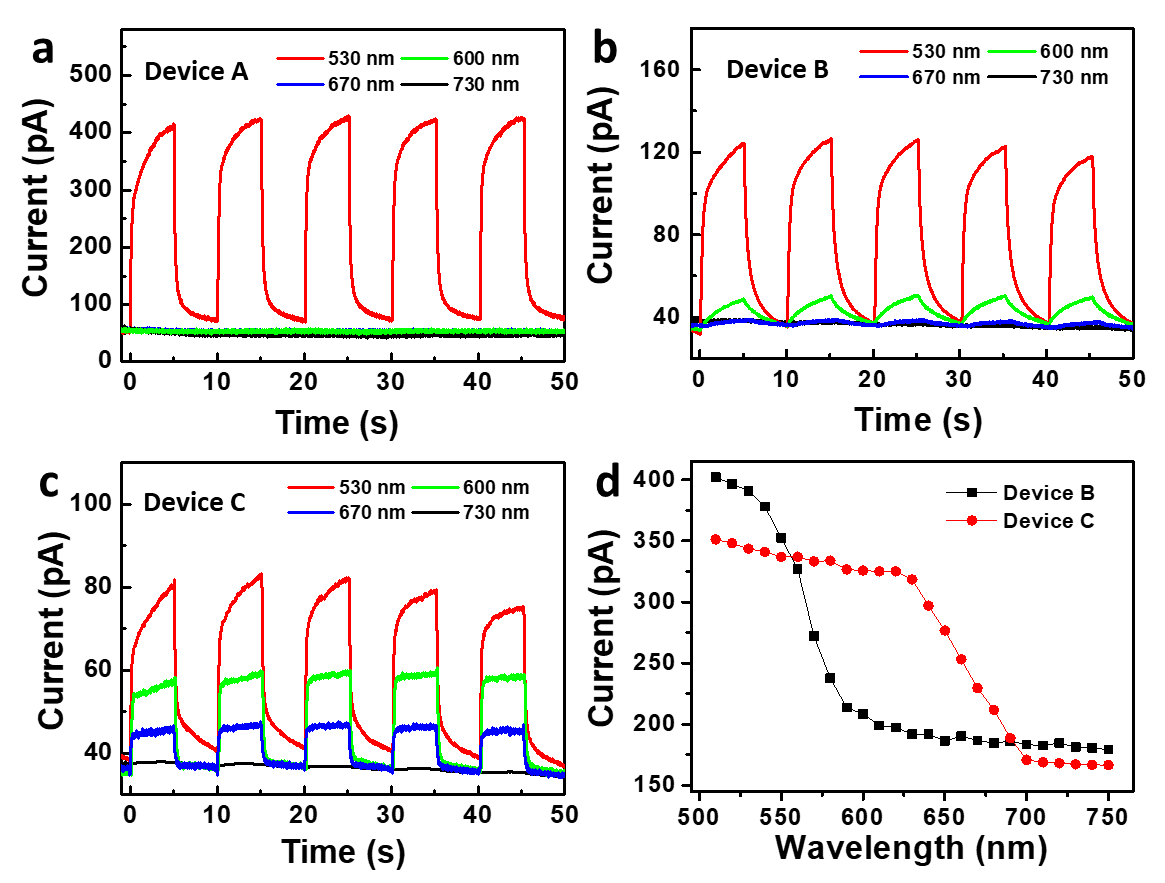
**

**3. Optoelectronic characteristics of the rainbow perovskite and heterojunction devices**

Fig. S5a shows an array of devices numbered from 1 to 10, which employ the Br/Cl or Br/I rainbow perovskites with a *L_GBG_* of around 4 mm as photosensing and conducting layers. For Device 6, the current shows an abrupt increase when the light wavelength decreases from 550 to 500 nm, and a similar phenomenon is also observed for Device 10 with reducing the light wavelength from 700 to 650 nm (Fig. S5b, c). The current versus time (*I-t*) curve of Device 6 shows typical rise and fall behaviors under pulse light with 500 nm wavelength, while it almost remains flat under 550 nm pulse light (Fig. S5d). Further, Fig. S5e presents the *I-t* curves of various devices under different wavelength pulse lights with a constant power density, in which all the devices exhibit cutoff responses to wavelength, and the “cutoff points” of Device 1, 3, 6, 8 and 10 are around 470, 500, 540, 630 and 690 nm, respectively. Apparently, the “cutoff points” to wavelength are determined by the bandgaps of the perovskite employed by the devices, and gradient-varying “cutoff points” of the devices further reveal that the perovskites at the corresponding locations possess gradient bandgaps. On the other hand, the devices with perovskites undergone dipping process exhibit rather low currents (Device 1, 3, 8 and 10 in Fig. S5e). C8BTBT is then introduced to construct heterojunction devices with the rainbow perovskites. Fig. S6a shows a fluorescence microscope photo of three heterojunction devices named as Device A, B and C, in which Device A is on the initial CsPbBr_3_ film while Device B and C are located on different positions of the Br/I GBG area with a *L_GBG_* of around 2 mm. The highest unoccupied molecular orbitals of both the perovskites and C8BTBT match with each other (Fig. S6b), which ensures the photogenerated holes in the perovskites can be transferred to the p-type C8BTBT. Accordingly, higher currents are obtained from the heterojunction devices as compared with the pure perovskite-based ones, and the photoresponse of the heterojunction devices comes from the perovskites instead of C8BTBT (Fig. S7). Fig. S8a-c present *I-t* curves of Device A, B and C under circular switching lights with different wavelengths, and the “cutoff points” to wavelength shifts from 530, 600 to 670 nm for the three devices, respectively. Current versus wavelength (*I-λ*) curves under different monochromatic lights with a constant power density are plotted for Device B and C (Fig. S8d). Significantly, the *I-λ* curves exhibit rather broad monotone decreasing ranges (*i.e.*, small slopes) in the initial “cutoff points”, *e.g.* the monotone decreasing range for Device B is from 520 to 600 nm, and that for Device C is from 630 to 700 nm. Such results are attributed to that a smaller *L_GBG_* leads to a broader range of varying bandgaps occupied by a single device, hence giving rise to more gradient changes for the wavelength-responsive curve.

Fig. S9. *I-λ* curve changes of the GBG device a) after light-switching cycles (with different light intensities), b) under an applied bias and c) after being stored in atmosphere for different times.

**
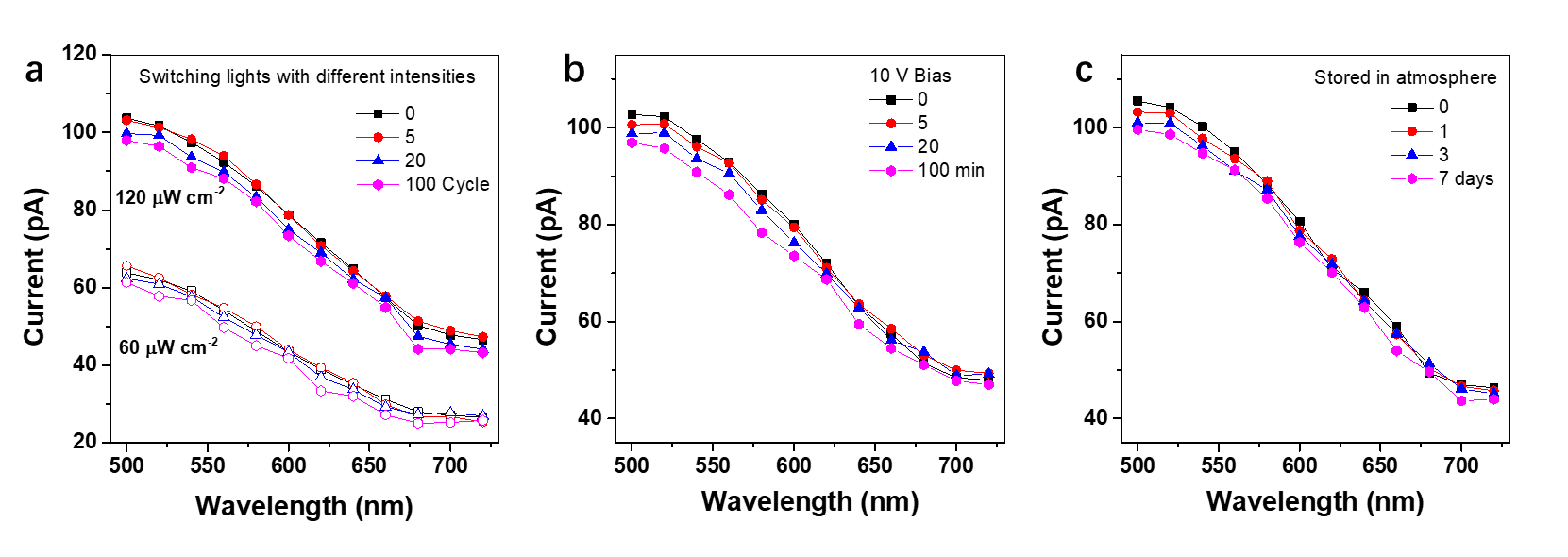
**

**Fig. S10.** Photon energy-responsive performance of the parallel and orthogonal heterojunction devices. a) Schemes of the heterojunction devices with different electrode directions, namely parallel and orthogonal devices. b) *I-λ* curves of the parallel and orthogonal devices (5 V, 0.1 mW cm^-2^).


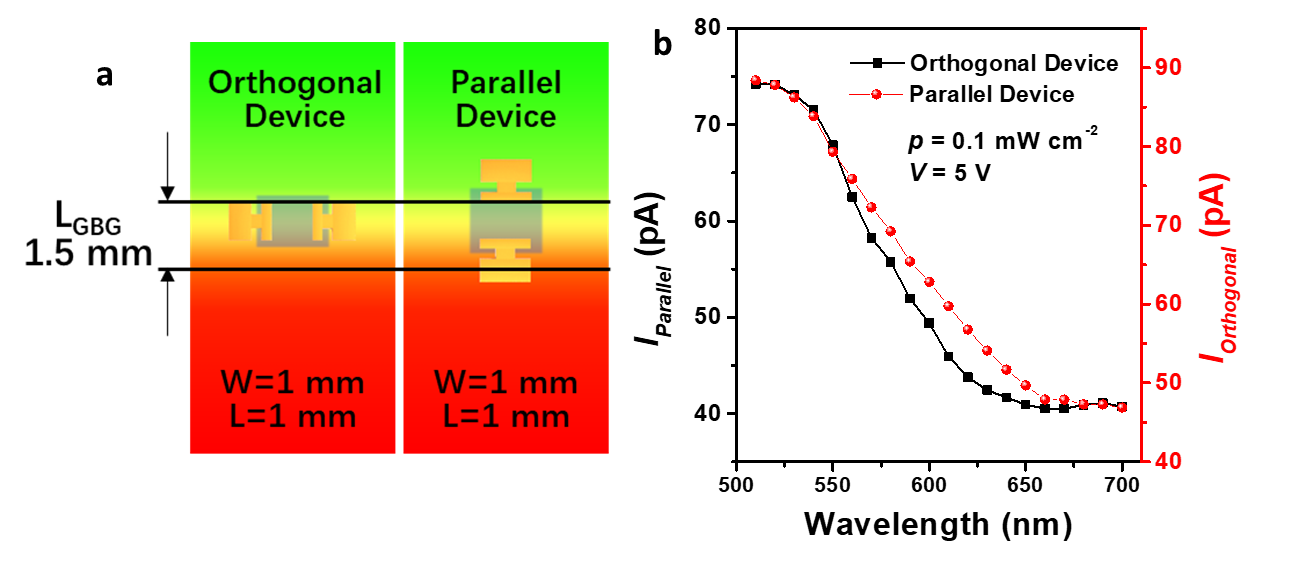


**Fig. S11.** a) Scheme of the device modelling for Model I. Variation of *ε_b_* as a function of *x_i_* or *y_i_* and the corresponding fitted lines for b) Device B and C and c) Device Ⅰ, Ⅱ and Ⅲ. Experimental and theoretical effective conductivity (*σ**) obtained from Model Ⅰ as a function of *ε* for d) Device B and C and e) Device Ⅰ, Ⅱ and Ⅲ.


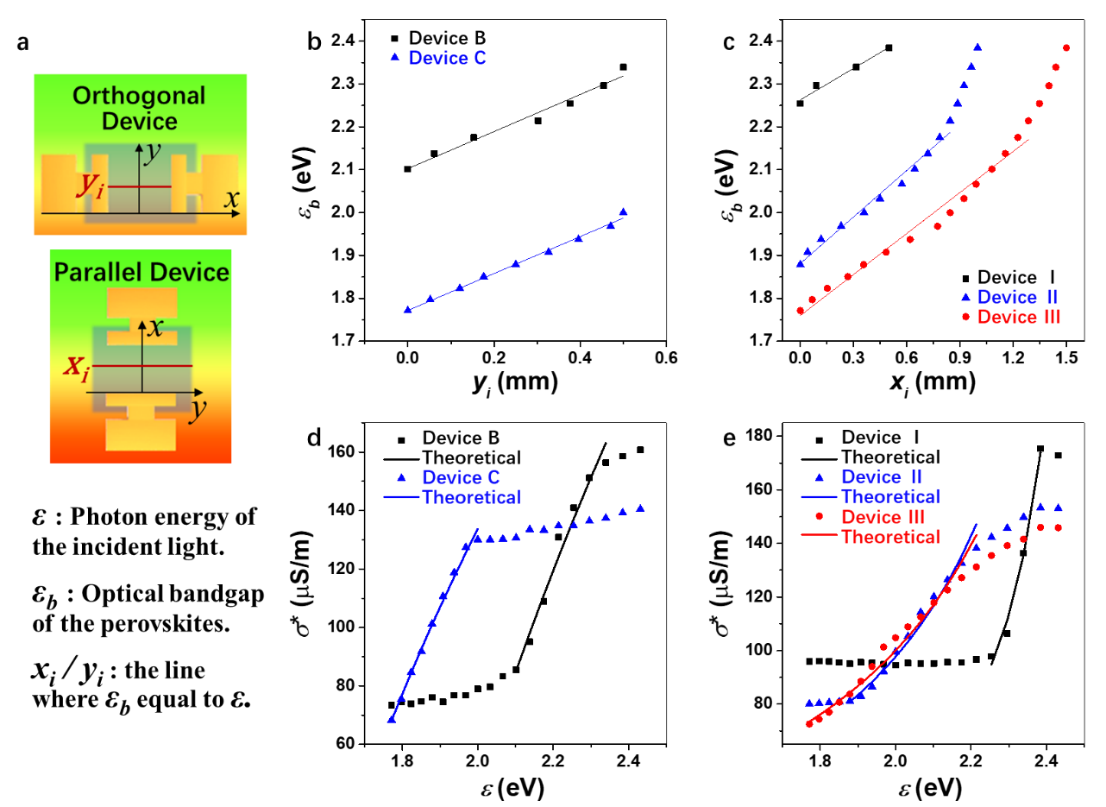


**4. Physical models of the GBG devices**

A mesoscopic volume is considered in the model, which is larger than the grain size of the perovskite and C8BTBT films (hundreds of nanometers), but smaller than the electrode intervals of the heterojunction devices (500 - 1500 μm). Assuming that the resistivity of the heterojunction devices comes from the grains more than the junctions, we have:

$j=\sigma E$ (S1)

where *j* is the current density, *σ* is the conductivity and *E* is the electric field. The conductivity depends on the charge *e_i_*, mobility *μ_i_* and density *n_i_* of each type of charge carrier *i*:

$\sigma=\sum_{i} \mu_{i}e_{i}n_{i}$ (S2)

Next, we assume that: (1) the conductivity is determined mainly by one type of the charge carrier (*e.g.* electron or hole), and (2) charge carriers can be split into dark (*n_d_*) and photogenerated ones (*n_p_*), so that we have $n=n_{d}+n_{p}$. The net growth rate of photogenerated charge carrier density can be given by:

$\frac{dn}{dt}=S-k_{a}n_{p}$ (S3)

where *S* and *k_a_* is the generation rate and the decay coefficient of photogenerated charge carriers, respectively. The density of photoexcited charge carriers can decay by three mechanisms, namely defects, bimolecular and Auger effect. At equilibrium, photogenerated charge carriers can be given by $n_{p}=n-n_{d}=\frac{S}{k_{a}}$. For a monochromatic light with photon energy *ε*, the photogeneration rate of charge carriers is also expressed by $S=\eta\left( \varepsilon\right)\varphi\left( \varepsilon\right)$, with $\eta\left( \varepsilon\right)$ the quantum efficiency and $\varphi\left( \varepsilon\right)$ the absorbed photon ﬂux. Given a direct bandgap for perovskites, the quantum efficiency can be approximately expressed by a step function_:_

$\eta=\eta_{0}H\left( \varepsilon-\varepsilon_{b} \right)$ (S4)

where *ε_b_* is the optical bandgap of the rainbow perovskites, and *H* is the Heaviside step function.

**(1) Model Ⅰ**

Firstly, we consider the photogenerated charge carriers in the perovskite film immediately transfer into the top C8BTBT layer, and the charge carrier density at the thickness direction is even. Then, the performance of the orthogonal and parallel devices can be different:

(a) For orthogonal devices (Device B and C)

Considering the current direction as *x*, the bandgap gradient direction is thus orthogonal to *x* (Fig. S11a). The charge conservation reads $\partial_{x}j=0$, so that $E_{x}=-\partial_{x}V$ is a constant. We immediately get $j=\sigma\frac{U}{L}$ with $U=V\left( 0 \right)-V\left( L \right)$*,* which yields the total electric current:

$I=\iint jdS=\frac{U}{L}\int_{y} \int_{z} \sigma dydz=\frac{hU}{L}\int_{0}^{W} \sigma_{h}dy$ (S5)

where *I* and *U* are the current and voltage, *σ_h_* represents the mean sheet conductivity, *h* is the thickness of the film, *L* and *W* is the length and width of the conducting film employed by the device, respectively. Substituting Equation S1 - S4 in Equation S5 yields:

$I=U\frac{h}{L}\int_{0}^{W} \mu e\left[ n_{d}+\frac{\varphi\eta_{0}}{k_{a}}H\left( \varepsilon-\varepsilon_{b} \right) \right]dy$ (S6)

Introducing *y_i_* as illustrated in Fig. S11a, the domain of the device larger than *y_i_* cannot be excited by the incident light. we recast the integral:

$I=U\frac{\mu en_{d}h}{L}\left[ \int_{0}^{y_{i}} \left( 1+\frac{\varphi\eta_{0}}{k_{a}n_{d}} \right)dy+\int_{y_{i}}^{W} 1dy \right]$ (S7)

which immediately yields:

$I=U\frac{\mu en_{d}h}{L}(W+\frac{\varphi\eta_{0}}{k_{a}n_{d}}y_{i})$ (S8)

Defining terms: dark conductivity $\sigma_{d}=\mu en_{d}$, and photocurrent gain $\xi=\frac{\eta_{0}}{k_{a}n_{d}}$, we can estimate the admittance of the device:

$G=\frac{\sigma_{d}h}{L}\left( W+\xi\varphi y_{i} \right)$ (S9)

Here, we consider the gradient of the varying bandgap is uniform (linear along *y* direction):

$\varepsilon_{b}=\varepsilon_{0}+ky_{i}$ (S10)

where *k* is the degree of the bandgap gradient. At *y_i_*, $\varepsilon=\varepsilon_{b}$. Then the eﬀective conductivity $\sigma^{*}$ is introduced as $\sigma^{*}=\frac{G\cdot L}{h\cdot W}$ , and we rearrange Equation S9:

$\sigma^{*}={\frac{\xi\sigma_{d}}{kW}\left( \varepsilon-\varepsilon_{0} \right)\varphi+\sigma}_{d}$ (S11)

(b) For parallel devices (Device Ⅰ, Ⅱ and Ⅲ)

Considering the bandgap gradient direction as *x*, and the current direction is parallel to *x* (Fig. S11a). we get $\partial_{x}j=0$ and $U=\int\frac{j}{\sigma}dx$, which yields the total voltage depending on the current:

$U=\frac{I}{Wh}\int_{0}^{L} \frac{1}{\sigma_{h}}dx$ (S12)

Substituting Equation S1 - S4 in Equation S12 yields:

$U=\frac{I}{Wh}\int_{0}^{L} \frac{1}{\mu e}\left[ n_{d}+\frac{\varphi\eta_{0}}{k_{a}}H\left( \varepsilon-\varepsilon_{b} \right) \right]^{-1}dx$ (S13)

Introducing *x_i_* as illustrated in Fig. S11a, the domain of the device larger than *x_i_* cannot be excited by the incident light. we have:

$U=I\frac{1}{Wh\mu en_{d}}\left[ \int_{0}^{x_{i}} \left( 1+\frac{\varphi\eta_{0}}{k_{a}n_{d}} \right)^{-1}dx+\int_{x_{i}}^{L} 1dx \right]$ (S14)

which immediately yields:

$U=I\frac{1}{Wh\mu en_{d}}\left[ \frac{x_{i}}{1+\frac{\varphi\eta_{0}}{k_{a}n_{d}}}+\left( L-x_{i} \right) \right]$ (S15)

Introducing terms $\sigma_{d}$ and $\xi$, we can estimate the resistance of the device:

$R=\frac{1}{Wh\sigma_{d}}\left( L-\frac{\xi\varphi x_{i}}{1+\xi\varphi} \right)$ (S16)

Similarly, we consider the gradient of the varying bandgap is uniform (linear along *x* direction):

$\varepsilon_{b}=\varepsilon_{0}+kx_{i}$ (S17)

At *x_i_,* $\varepsilon=\varepsilon_{b}$. Then $\sigma^{*}$is introduced as $\sigma^{*}=\frac{L}{R\cdot W\cdot h}$, and we recast Equation S16 into:

$\sigma^{*}=\sigma_{d}\frac{1+\xi\varphi}{1+\xi\varphi\left( 1-\frac{\varepsilon-\varepsilon_{0}}{kL} \right)}$ (S18)

(c) Parameters fitting

Now, the parameters of the devices, including Device B, C, Ⅰ, Ⅱ and Ⅲ, can be obtained by fitting the experimental *σ*-ε* curves. *ε_0_* and *ε_1_* are the beginning and end points of the quasi-linear regions for the *σ*-ε* curves with constant *p*, respectively. $\sigma_{d}$ are found for the devices under lights with the photon energy *ε* = *ε_0_*, that is, *x_i_* = *y_i_* = 0. This gives$G\left( \varepsilon_{0} \right)=\frac{\sigma_{d}h}{L}W$ and $R\left( \varepsilon_{0} \right)=\frac{1}{Wh\sigma_{d}}L$. At the photon energy *ε* = *ε_1_* (*x_i_* = *L* and *y_i_* = *W*), *ξ* can be calculated. *y_i_*-*ε_b_* or *x_i_*-*ε_b_* data for the five devices can be obtained by recasting Equation S9 and S16, as at *y_i_* or *x_i_,* $\varepsilon=\varepsilon_{b}$. Then, the bandgap gradient *k* was obtained by linear fitting the *y_i_*-*ε_b_* and *x_i_*-*ε_b_* curves as shown in Fig. S11b and c. Finally, theoretic *σ** can be calculated from Equation S11 and S18. All the fitted parameters are shown in Table S1, and the theoretic *σ** are presented in Fig. S11d and e as compared with the experimental ones. The values of two kinds of *σ** are consistent with each other in many cases. However, for Device Ⅱ and Ⅲ under incident lights with rather high photon energies, the model becomes less accurate (Fig. S11d and e).

**Table S1**. The fitted parameters of the heterojunction devices according to Model Ⅰ.

| Device | *L*  (mm) | *W*  (mm) | *ε*_0_  (eV) | *ε*_1_  (eV) | $\sigma_{d}$  (μS m^-1^) | *ξ*  (10^-15^ s⋅cm^2^) | *k*  (eV⋅mm^-1^) |
| --- | --- | --- | --- | --- | --- | --- | --- |
| B | 0.1 | 0.5 | 2.10 | 2.34 | 85.49 | 3.22 | 0.43 |
| C | 0.1 | 0.5 | 1.77 | 2.00 | 68.32 | 3.45 | 0.43 |
| Ⅰ | 0.5 | 2.0 | 2.25 | 2.38 | 97.80 | 3.30 | 0.25 |
| Ⅱ | 1.0 | 2.0 | 1.88 | 2.38 | 81.20 | 2.95 | 0.36 |
| Ⅲ | 1.5 | 2.0 | 1.77 | 2.38 | 72.52 | 2.59 | 0.32 |

**Fig. S12.** Plotting of *σ** versus *ε* for Device B and C under a constant a) light power density (0.1 mW/cm^2^) and b) photo flux (3.4×10^14^ s^-1^cm^-2^) together with fitted lines according to Model Ⅱ.

**
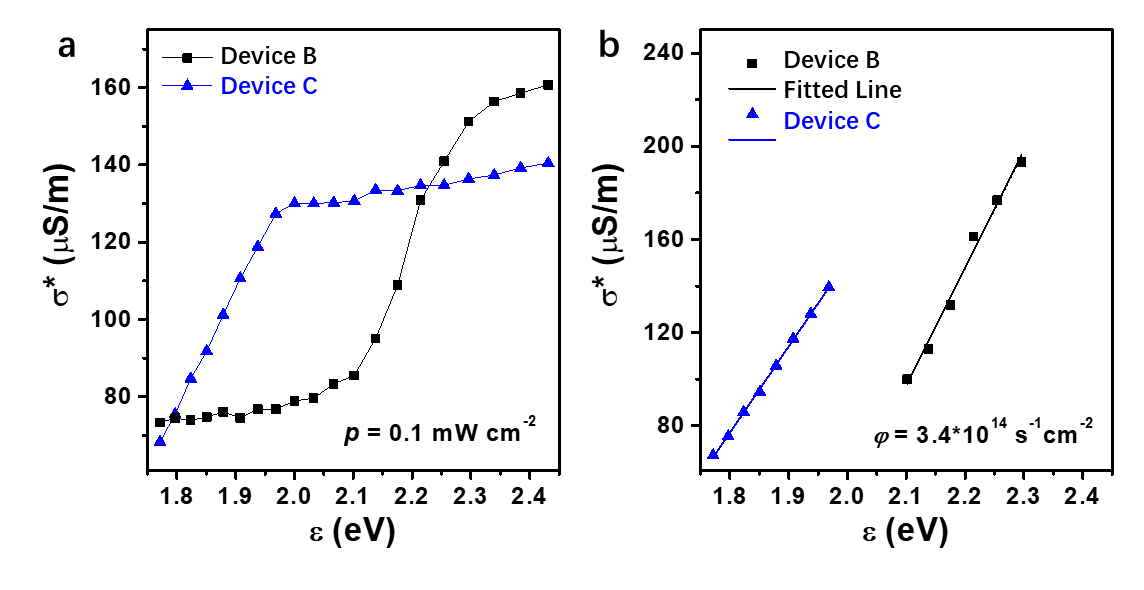
**

**(2) Model Ⅱ**

Then, we consider the transferred photoexcitons from the perovskite film uniformly distribute in the C8BTBT film immediately, so that the photogenerated charge carrier density is even at the film plane. In this case, performance of the parallel and orthogonal device can be similar. We define the direction of bandgap gradient as *x*, and the length of the GBG film employed by the device is *l* (no matter parallel or orthogonal device). Given the GBG film is uniform at width and thickness direction, the mean photogenerated charge carrier density in the conducting layer transferred from the rainbow perovskite can be expressed as:

$n_{p}=\frac{{\varphi\eta}_{0}}{k_{a}}\frac{\int_{0}^{l} H\left( \varepsilon-\varepsilon_{b} \right)dx}{l}$ (S19)

Substituting Equation S1 - S4 into Equation S19, the effective conductivity of the device:

$\sigma^{*}=\mu e\left[ n_{d}+\frac{{\varphi\eta}_{0}}{lk_{a}}\int_{0}^{l} H\left( \varepsilon-\varepsilon_{b} \right)dx \right]$ (S20)

Introducing *x_i_* as illustrated above, the domain of the device larger than *x_i_* cannot be excited by the illumination. we have:

$\sigma^{*}=\mu en_{d}\left( 1+\frac{{\varphi\eta}_{0}}{lk_{a}n_{d}}\int_{0}^{x_{i}} 1dx \right)$ (S21)

which immediately yields:

$\sigma^{*}=\frac{\mu en_{d}}{l}(l+\frac{{\varphi\eta}_{0}}{k_{a}n_{d}}x_{i})$ (S22)

Introducing terms $\sigma_{d}$ and $\xi$, and we consider the gradient of the varying bandgap is uniform:

$\varepsilon_{b}=\varepsilon_{0}+kx_{i}$ (S23)

At *x_i_,* $\varepsilon=\varepsilon_{b}$, which eventually gives:

$\sigma^{*}={\frac{\xi\sigma_{d}}{kl}\left( \varepsilon-\varepsilon_{0} \right)\varphi+\sigma}_{d}$ (S24)

Such equation is the same with Equation S11 in Model Ⅰ for the orthogonal devices. The experimental *σ*-ε* curves with fixed *φ* can be linearly fitted perfectly with the Equation S24, as shown in Fig. 3f and Fig. S12. According to Model Ⅱ, it is also well explained that the electric current direction, either orthogonal (Device B and C) or parallel (Device Ⅰ, Ⅱ and Ⅲ), exhibits limited effect on the device performance (Fig. S12). Therefore, we think Model Ⅱ is more accurate than Model Ⅰ to describe the heterojunction devices. Now, the parameters in the Equation S24 for different devices can be obtained. $\sigma_{d}$ are found for the devices in the dark, while *ξ* is found for the devices under lights with the shortest wavelengths. *k* and *ε_0_* are obtained by linearly fitting the *σ*-ε* curves. The fitted parameters are shown in Table S2, which is consistent with experimental results. The dark conductivity of Device B is higher that of Device C, and from Device Ⅰ to Ⅲ, the dark conductivity keeps decreasing. Such tendency is because the dipping process reduces the conductivity of the perovskites. The calculated *ε_0_* for the 5 devices is consistent with their locations on the GBG films, respectively. If the gradient of the varying bandgap is ideally linear, *k* for Device B and C should be the same and equal to (*ε_CsPbBr3_*-*ε_CsPbI3_*)/*L_GBG_* around 0.31 eV⋅mm^-1^, while those for Device Ⅰ, Ⅱ and Ⅲ should be around 0.41 eV⋅mm^-1^. However, the obtained *k* values are discrepant around the ideal ones, indicating that the uniformity of the bandgap gradients of the rainbow perovskites can be further improved.

**Table S2**. The fitted parameters for the heterojunction devices according to Model Ⅱ.

| Device | *l*  (mm) | $\sigma_{d}$  (μS m^-1^) | *ξ*  (10^-15^ s⋅cm^2^) | *ε_0_*  (eV) | *k*  (eV⋅mm^-1^) |
| --- | --- | --- | --- | --- | --- |
| B | 0.5 | 73.3 | 4.77 | 2.05 | 0.47 |
| C | 0.5 | 68.3 | 3.06 | 1.78 | 0.38 |
| Ⅰ | 0.5 | 95.1 | 4.25 | 2.24 | 0.32 |
| Ⅱ | 1 | 80.1 | 4.53 | 1.87 | 0.49 |
| Ⅲ | 1.5 | 72.5 | 4.92 | 1.79 | 0.39 |

**Fig. S13.** A structural scheme of the flexible heterojunction device.


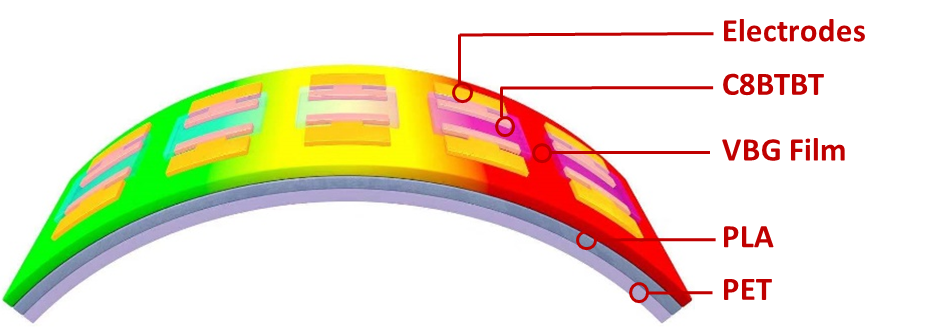


**5. Calibration of the GBG device**

The photogenerated currents of the GBG device under given voltages can be expressed as:

$I_{ph}=a\left( \varepsilon-\varepsilon_{0} \right)\varphi+b$ (S25)

and the parameters *a*, *b* and *ε_0_* can be obtained from the calibrations. According to the linearly fitted lines in Fig. 4c, we have:

$I_{ph}=3.6{10}^{-15}\cdot\varphi+13$ (S26)

when *ε* equal to 2.34 eV, and

$I_{ph}=277.2\cdot\varepsilon-507$ (S27)

when *φ* equal to 3.4×10^14^ S^-1^cm^-2^. Combining Equation S25 - S27, we have *a* = 81.5×10^-14^, *b* = 13 and *ε_0_* = 1.88 eV (or 1.89 eV) for the heterojunction device. Therefore, $\overline{\varepsilon_{\varphi}}$ and *φ* of the incident radiations can be obtained by resolving the following equation set:

$\left\{ \begin{aligned} I_{ph}=81.5{10}^{-14}\left( \overline{\varepsilon_{\varphi}}-1.88 \right)\varphi+13 \\ p=\overline{\varepsilon_{\varphi}}\times\varphi\end{aligned} \right.$ (S28)

where the power density of each radiation is measured by a commercial light power density meter.

**6. Comparison of** $\overline{\boldsymbol{\varepsilon}_{\boldsymbol{\varphi}}}$ **and** $\bar{\boldsymbol{\varepsilon}_{\boldsymbol{p}}}$

The average photon energies weighted by the power density $\bar{\varepsilon_{p}}$ are obtained from the spectral curves in Fig. 4a by using the following equation:

(S29)

$$\bar{\varepsilon_{p}}=\frac{\sum_{i} {\varepsilon_{i}p}_{i}}{\sum_{i} p_{i}}$$

where the interval *i* is determined by the wavelength resolution of the spectrometer, and *p* is the normalized power density. Briefly speaking, $\overline{\varepsilon_{\varphi}}$ is weighted by *φ*, while $\bar{\varepsilon_{p}}$ is weighted by *p*. A larger *ε* gives rise to a larger *p* even for a constant *φ*, indicating that a larger *ε* possesses a larger weight in $\bar{\varepsilon_{p}}$. Therefore, the value of $\bar{\varepsilon_{p}}$ should be larger than that of $\bar{\varepsilon_{\varphi}}$, which is consistent with the experimental results, as shown in Fig. 4e. Nevertheless, the both values of $\bar{\varepsilon_{\varphi}}$ and $\bar{\varepsilon_{p}}$ are close to each other.
